# Aerobic exercise regulates FGF21 and NLRP3 inflammasome-mediated pyroptosis and inhibits atherosclerosis in mice

**DOI:** 10.1371/journal.pone.0273527

**Published:** 2022-08-25

**Authors:** Xiao-Hong Li, Liang-Zhong Liu, Lin Chen, Qi-Ni Pan, Zi-Yao Ouyang, De-Jing Fan, Xiao Pan, Su-Yu Lu, Qiu-Hu Luo, Pin-Yue Tao, Hui-Qiao Huang

**Affiliations:** 1 Department of Cardiology, The Second Affiliated Hospital of Guangxi Medical University, Nanning, Guangxi, China; 2 Department of Oncology, Chongqing University Three Gorges Hospital, Wanzhou, Chongqing, China; 3 Emergency Department, The Second Affiliated Hospital of Guangxi Medical University, Nanning, Guangxi, China; 4 Department of Anesthesiology, The Second Affiliated Hospital of Guangxi Medical University, Nanning, Guangxi, China; 5 Department of Cardiology, The First Affiliated Hospital of Guangxi Medical University, Nanning, Guangxi, China; University of Kentucky, UNITED STATES

## Abstract

Fibroblast growth factor 21 (FGF21), a known risk factor for atherosclerosis, is readily regulated by exercise, and it can inhibit NOD-like receptor protein 3 (NLRP3)-mediated pyroptosis. However, it is not clear whether aerobic exercise inhibits atherosclerosis via these pathways. Eight-week-old apolipoprotein E-deficient (*ApoE*^*-/-*^) mice on a high-fat diet were randomly divided into 1-h post-exercise (EX-1h), 24-h post-exercise (EX-24h), and sedentary (SED) groups. C57BL/6J wild-type mice fed normal chow served as controls (WT group). Mice in the EX-1h and EX-24h groups were subjected to treadmill exercise training for 12 weeks. Aerobic exercise reduced body weight; blood glucose, lipid, and inflammation levels; and aortic plaque area proportion. Aerobic exercise increased the sensitivity of FGF21 by upregulating the expression of the downstream receptor adiponectin (ApN); the serum FGF21 level after exercise increased initially, and then decreased. Aerobic exercise downregulated the expression of NLRP3 inflammasome-mediated pyroptosis-related markers in the aorta, and FGF21 may participate in the above process. Meanwhile, the liver may be the tissue source of serum FGF21 during aerobic exercise. In conclusion, aerobic exercise may inhibit atherogenesis by regulating FGF21 and NLRP3 inflammasome-mediated pyroptosis. Our study provides new information on the atherosclerosis-preventing mechanism of aerobic exercise.

## Introduction

Atherosclerosis, a chronic inflammatory disease associated with high morbidity and mortality rates [1], is characterized by increased cell death and inflammatory factor release [2, 3]. Lipid metabolism disorders and inflammation promote the formation of atherosclerotic plaques in arterial walls. Aerobic exercise has been shown to reduce body weight; normalize blood glucose, lipid, and inflammatory levels; and enhance antioxidant capacity. This is an important strategy to prevent atherosclerosis [4–6]. However, the mechanisms by which aerobic exercise prevents atherosclerosis have not been fully elucidated.

Pyroptosis is a programmed inflammatory cell death process. The development of atherosclerosis involves the pyroptosis of endothelial cells, macrophages, and smooth muscle cells [7]. The occurrence of pyroptosis depends on the activation of inflammasomes and cysteinyl aspartate-specific proteinases (caspases). NOD-like receptor protein 3 (NLRP3) inflammasome is the most representative and easily recognized inflammasome [8]. The classical pyroptosis pathway mediated by the NLRP3 inflammasome is involved in the occurrence and development of atherosclerosis [9]. In this pathway, NLRP3 recruits the apoptosis-associated speck-like protein containing a caspase recruitment domain (ASC) through homotypic interactions, and the ASC recruits pro-caspase-1 to form the inflammasome. Pro-caspase-1 cleaves itself and forms mature, activated caspase-1 [10]. Activated caspase-1 cleaves and activates Gasdermin D (GSDMD), which can lead to membrane pore formation. Caspase-1 also cleaves pro-IL-1β and pro-IL-18 into mature IL-1β and IL-18 [11, 12], which are released from the cell through membrane pores, leading to inflammation and cell death [13]. Pyroptosis is easily regulated. Negative regulation of the NLRP3 inflammasome to inhibit pyroptosis is an important research direction for the prevention and treatment of atherosclerosis [14, 15].

Fibroblast growth factor 21 (FGF21), a multifunctional protein, is a member of the FGF19 subfamily. FGF21 is mainly secreted by the liver, adipose tissues, pancreas, and muscle, and is also expressed in the aorta, and serum FGF21 is mainly secreted by the liver [16–18]. FGF21 has the functions of reducing blood glucose and lipid levels, restoring mitochondrial function, and weakening oxidation stress injury [19–21]. FGF21 can also inhibit endothelial cell pyroptosis mediated by NLRP3 inflammasome and reduce aortic pyroptosis mediated by the NLRP3 inflammasome to prevent atherosclerosis [22–24]. Deficiency of FGF21 aggravates the development of atherosclerosis [25]. Paradoxically, the serum FGF21 level is elevated in patients with atherosclerosis [26] and is one of the most sensitive predictors of atherosclerosis [27]. Studies have shown that the downstream signal passage of FGF21 in patients with atherosclerosis is damaged, resulting in FGF21 resistance [28, 29]. This indicates that more FGF21 is needed than physiological doses to meet the physiological needs [30]. Therefore, reducing FGF21 resistance and improving its sensitivity may be an approach to prevent atherosclerosis.

Exercise is known to regulate the level of serum FGF21 [31], but its increase or decrease is controversial. Serum FGF21 level significantly increases after 2 weeks of exercise [32], but drops after 36 weeks of exercise in patients with obesity and after 12 weeks in obese rats [33, 34]. It has been shown that the effects of exercise on the serum FGF21 level are time dependent, and the serum FGF21 level peaks at 1 h after exercise, and then starts to decrease [17, 35]. Therefore, the contradictory results of previous studies may be related to different blood collection times. Further studies have revealed that the serum FGF21 level decreased after 36 or 12 weeks of aerobic exercise, but triglyceride (TG) level and body mass index (BMI) decreased, and the arterial stiffness is reduced in the study subjects [33, 34]. The above findings suggest that the **s**ensitivity of FGF21 may be increased after aerobic exercise, but further research is needed in this regard. However, there are limited data on whether aerobic exercise modulates FGF21 to inhibit pyroptosis mediated by NLRP3 inflammasome and then prevent atherosclerosis. In this study, we established an atherosclerosis mouse model by feeding a high-fat diet. We observed the effects of aerobic exercise on FGF21 and NLRP3 inflammasome-mediated pyroptosis, and serum FGF21 level change trend after aerobic exercise. In addition, the tissue origin of serum FGF21 during aerobic exercise was explored. Our study might provide new information on the atherosclerosis-preventing mechanism of aerobic exercise.

## Materials and methods

### Experimental animals

Specific pathogen-free C57BL/6J *ApoE*^*-/-*^ (n = 60) and wild-type C57BL/6J (n = 20) male mice, all 8 weeks old, were purchased from Beijing Vital River Laboratory Animal Technology Co., Ltd. (animal quality qualified license number: SCXK (Beijing) 2016–0006). C57BL/6J mice were fed a regular mouse diet whereas *ApoE*^*-/-*^ mice were provided a high-fat diet containing 20% lipid and 1.5% cholesterol [36]. All mice were maintained under a 12-h light–dark cycle (lights on at 8:00 am, lights off at 8:00 pm) at 22°–24°C and 50%–55% humidity, with free access to food and water. After a week of adaptation to the environment, *ApoE*^*-/-*^ mice were randomly divided into the following three groups of 20 mice each: 1-h post-exercise training (EX-1h), 24-h post-exercise training (EX-24h), and sedentary (SED) groups. Wild-type C57BL/6J mice served as healthy controls (WT; n = 20). Food intake and body weight of mice were monitored on a weekly basis. Mice in the SED and WT groups were restricted from freely moving in the cage. The study was conducted according to the Guidelines for the Care and Use of Laboratory Animals of the National Institutes of Health (NIH) and approved by the Animal Care and Welfare Committee of Guangxi Medical University (202010021; October 10, 2020).

### Exercise protocol

Mice in the WT and SED groups were restricted to move freely in their cages throughout the experiment. Mice in the EX-1h and EX-24h groups ran on a treadmill at a constant pace for 12 weeks starting at 9:00 AM (5 days of exercise and 2 days of rest per week), and the rest of the time was restricted to move freely in the cages same as the mice in the WT and SED groups. The exercise programs of EX-1h and EX-24h groups were the same and were determined according to previous studies [37–39]. Speeds and durations were as follows: week 1: 10 m/min, 30 min/day; week 2: 12 m/min, 60 min/day; weeks 3–12: 15 m/min, 60 min/day. This level of exercise is equivalent to 60%–80% of the maximum oxygen consumption [37].

### Tissue samples

Sampling was performed 24 h after the last exercise period, with 12 h of fasting. All mice were euthanized by sodium pentobarbital (50 μg/g/BW) and sacrificed for the further study. Blood was collected from the ocular venous plexus; the needle of the infusion set was inserted from the apex of the heart into the left ventricle and phosphate-buffered saline was injected at a constant rate to flush the aorta. The aorta and right lobe of the liver were excised. Mice in the EX-1h group were made to exercise again 22 h after the last exercise (unchanged at 15 m/min for 60 min). Samples were collected one hour after exercise.

### Blood analysis

Commercial kits were used to measure triglyceride (TG), total cholesterol (TC), low-density lipoprotein-cholesterol (LDL-C), high-density lipoprotein-cholesterol (HDL-C), free fatty acids (FFAs), and glucose levels in serum (Servicebio, Wuhan, China). We measured blood levels of FGF21 (R&D Systems, Minneapolis, MN, USA), IL-1β (R&D Systems), IL-18 (R&D Systems), and adiponectin (Cusabio, Wuhan, China) using commercially available enzyme-linked immunosorbent assay (ELISA) kits. All assays were performed according to the manufacturers’ instructions.

### Aortic lesion assessment

Oil red O staining and hematoxylin and eosin (H&E) staining were performed to evaluate the formation of aortic plaque. From each experimental group, five entire aortas were cut longitudinally and stained with Oil Red O. A high-resolution camera was used to capture images of the aorta; the images were analyzed using ImageJ software to evaluate the percentage of plaque area. Five aortic roots from each group were embedded in paraffin and cut transversely into 5-μm thick sections, which were stained with H&E. We observed and collected images using a microscope to analyze the morphology of the blood vessels. All samples were analyzed using a blinded method.

### Real-time polymerase chain reaction (RT-PCR)

Glass homogenizers were used to homogenize the aortas and livers. The TRIzol Total RNA Extraction Kit (Takara, Tokyo, Japan) was used to extract RNA from the homogenates. A spectrophotometer was used to determine RNA purity and concentration. The RNA was used as a template to synthesize cDNA by reverse transcription. The cDNA was used as a PCR template and *GAPDH* as the internal reference. SYBR^®^ Premix Ex Taq™ was used for PCRs, performed in an ABI StepOne Plus cycler (Applied Biosystems, California, USA). All reactions were carried out in triplicate, with three independent experiments. The 2^-ΔΔCT^ method was used to quantify mRNA. Primers (Table 1) were based on gene sequences from the GenBank database and were synthesized by Bao Biomedical Technology Co., Ltd. (Beijing, China).

**Table 1:** Forward/reverse (F/R) primer sequences.

Primer	Sequence (5′-3′)
FGF21-F	ACACTGAAGCCCACCTGGAGA
FGF21-R	CTGCAGGCCTCAGGATCAAAG
NLRP3-F	ACTGAAGCACCTGCTCTGCAAC
NLRP3-R	AACCAATGCGAGATCCTGACAAC
Caspase-1-F	ACTCGTACACGTCTTGCCCTCA
Caspase-1-R	CTGGGCAGGCAGCAAATTC
GSDMD-F	CGTTATTCATGTGTCAACCTGTCAA
GSDMD-R	TCCCATCGACGACATCAGAGA
IL-1β-F	TCCAGGATGAGGACATGAGCAC
IL-1β-R	GAACGTCACACACCAGCAGGTTA
IL-18-F	CCCTTTGAGGCATCCAGGAC
IL-18-R	TGGGAACAGCCAGTGTTCAG

### Western blotting

The aorta samples were homogenized in lysis buffer and total protein was extracted. A bicinchoninic acid kit was used to quantify proteins (Bio-Rad, Rockford, IL, USA). After dissolving 100 μg protein sample/well, proteins were separated using 9% SDS-PAGE and transferred onto polyvinylidene fluoride membranes. The membranes were blocked with skim milk (5%, dissolved in phosphate-buffered saline) for 2 h. Primary antibodies against the following antigens were added: FGF21 (1:1000; Abcam, Cambridge, UK), NLRP3 (1:1000; Abcam), caspase-1 (1:1000; CST, Boston, MA, USA), GSDMD (1:1000; Abcam), IL-1β (1:1000; Abcam), and GAPDH (1:10000; Abcam); the blots were incubated overnight at 4°C. The blots were incubated with horseradish peroxidase-labeled goat anti-rabbit IgG as the secondary antibody (1:5000; Calbiochem, San Diego, CA, USA) for 1 h at 20°C–25°C, washed thrice with TBS-T, developed using an ECL luminescence kit, and imaged. ImageJ software was used to analyze the protein bands. GADPH was used as an internal reference.

### Immunofluorescence

The aortic root and liver samples were subjected to immunofluorescence staining to observe aortic NLRP3 and FGF21 expression and liver FGF21 expression. Paraffin-embedded tissue blocks were cut into sections of thickness 5 μm. The sections were treated with EDTA antigen-retrieval solution for 5 min, blocked with 3% serum for 30 min, and then incubated overnight in a humidity chamber at 4°C with a primary antibody against FGF21 (1:200; Servicebio). Next, the sections were incubated at 20°C–25°C in the dark with fluorescent secondary antibody (CY3 goat anti-rabbit) for 50 min. TSA working solution was added (50 μL) on the sections, which were then incubated for 10 min and subjected to EDTA microwave treatment. Subsequently, the primary antibody against NLRP3 (1:200; Wanleibio, Liaoning, China) was added on the sections, which were then incubated overnight in a humidified box at 4°C. Fluorescent F488 goat anti-rabbit secondary antibody was added, and the sections were incubated for 50 min in the dark at 20°C–25°C. DAPI was used to counterstain the nuclei. The sections were subjected to quenching of tissue autofluorescence, mounting, and observation using a fluorescence microscope.

### Statistical analysis

Data are presented as mean ± SD. SPSS 22.0 was used for statistical analysis. A one-way ANOVA and Student–Newman–Keel *post hoc* multiple comparison tests were used to examine the experimental data. At *p* < 0.05, differences were considered statistically significant.

## Results

### Aerobic exercise training reduces body weight

**Fig 1:**
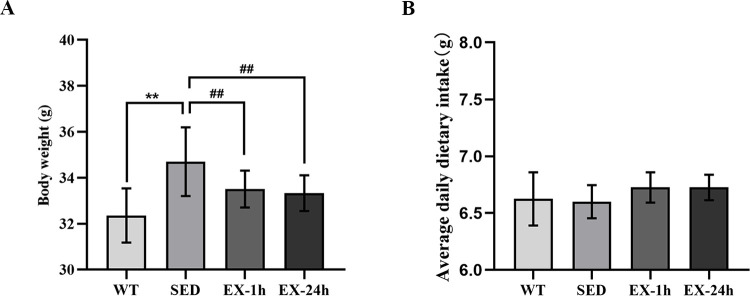
Aerobic exercise reduces body weight without changing dietary intake. (**A**) Body weight at the end of the experiment. (**B**) Average daily dietary intake. Data are presented as mean ± SD (n = 20). **p < 0.01 vs. WT group; ^##^ *p* < 0.01 vs. SED group.

Body weight and average weekly dietary intake of the mice were compared before and at the end of the experiment. At baseline (8 weeks of age), there were no significant differences in weight. At the end of the experiment, mice in the SED group were significantly heavier than those in the WT group (*p* < 0.01; 34.7 ± 1.5 g vs. 32.4 ± 1.2 g), whereas mice in the EX-1h and EX-24h groups were lighter than those in the SED group (*p* < 0.01; 33.5 ± 0.8 g and 33.3 ± 0.8 g vs. 34.7 ± 1.5 g; Fig 1A). There were no significant differences in the average daily dietary intake between the groups (Fig 1B).

### Aerobic exercise inhibits atherosclerosis

**Fig 2:**
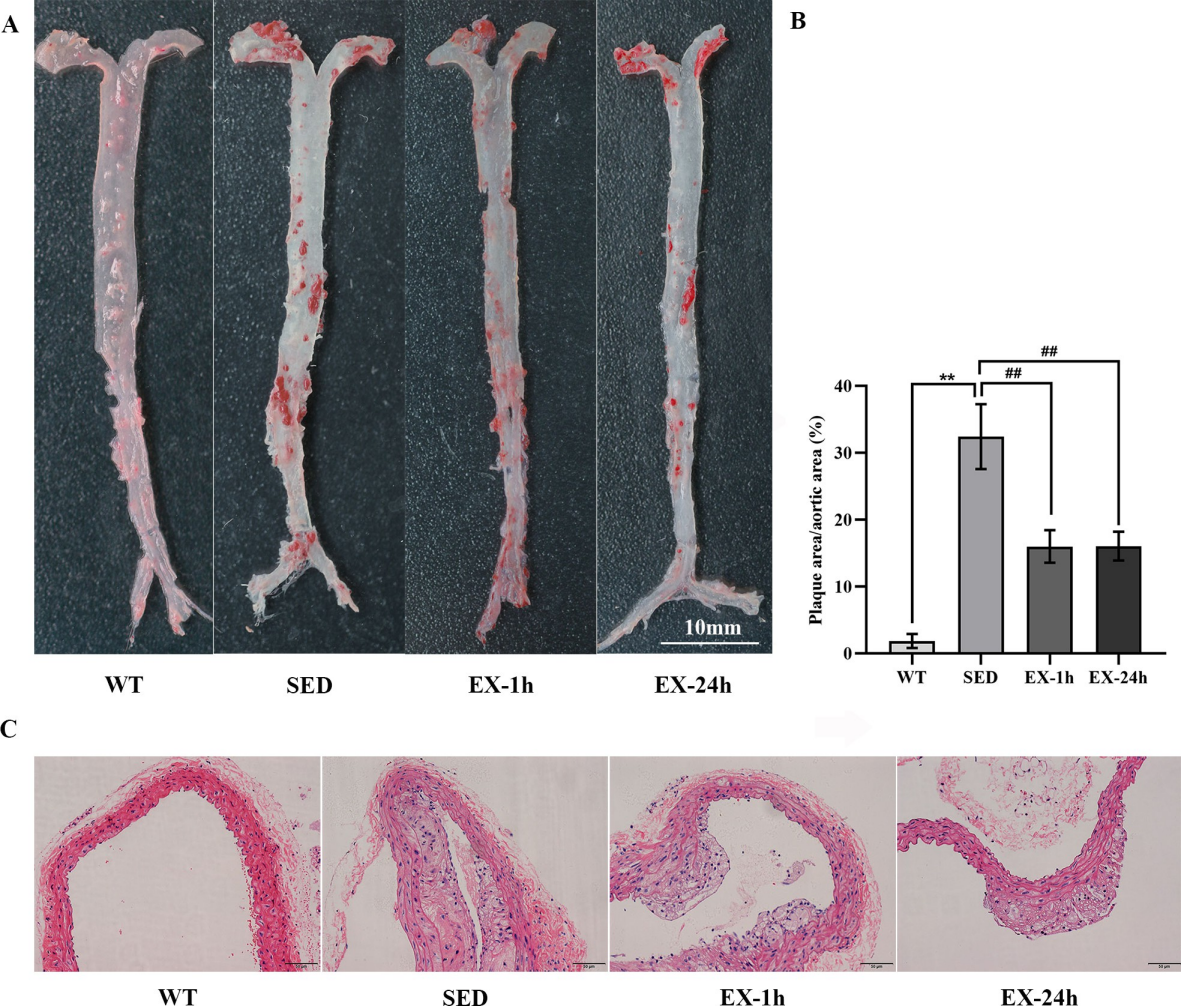
Aerobic exercise inhibits atherosclerosis. (**A**) Representative images of the whole aortic with oil red O staining. Scale bar = 10 mm; (**B**) Comparison of the proportion of aortic plaque area. Data are presented as mean ± SD (n = 5). **p <0.01 vs. WT group; ^##^ *p* <0.01 vs. SED group. (**C**) Representative images of hematoxylin and eosin (H&E) staining of cross-section of aortic root. Scale bar = 50 μm.

We performed Oil Red O staining of the entire aorta to compare the formation of aortic plaques in mice of each group (Fig 2A and 2B) and performed H&E staining on the cross-sections of the aortic root to observe the morphological changes (Fig 2C).

The aortas of the mice in the WT group exhibited almost no visible plaques. Those of the mice in the SED, EX-1h, and EX-24h groups displayed varying degrees of plaque formation (Fig 2A). The proportion of aortic plaque areas in the EX-1h and EX-24h groups was significantly smaller than that of the SED group (*p* < 0.01; 16.6% ± 2.3% and 16.1% ± 2.2% vs. 32.4% ± 4.8%; Fig 2B).

The vascular structure and morphology of the aortic root in the WT group were normal and intimal continuity was intact. In the SED group, there was obvious foam-cell aggregation and cholesterol crystal deposition under the intima, with large numbers of plaques almost filling the entire blood vessel lumen. In contrast, the extent of intra-aortic plaque lesions in the EX-1h and EX-24h groups were significantly reduced compared with the SED group (Fig 2C).

### Aerobic exercise reduces blood glucose, lipid, and inflammatory levels

We measured TC, TG, HDL-C, LDL-C, glucose, IL-1β, and IL-18 levels in all four groups (Table 2). Compared with the WT controls, the SED group had high blood glucose, lipid, and inflammatory levels (*p* < 0.05). Both EX-1h and EX-24h groups presented lower blood glucose, lipid, and inflammatory levels than the SED group (*p* < 0.05).

**Table 2:** Effects of aerobic exercise on blood glucose, lipids and inflammation levels.

	WT	SED	EX-1h	EX-24h	F	*P*
TG (mmol/L)	0.5±0.1	2.7±0.2**	1.0±0.1^##^	1.4±0.2^##^^,^ ^&&^	205.768	<0.001
TC (mmol/L)	2.8±0.3	17.3±2.5**	7.7±1.1^##^	14.7±1.8^##^^,^ ^&&^	112.858	<0.001
HDL-C (mmol/L)	1.9±0.3	1.6±0.2	2.0±0.2^##^	2.1±0.2	6.968	<0.001
LDL-C (mmol/L)	0.4±0.1	13.1±2.0**	7.6±1.1^##^	11.0±1.5^##^^,^ ^&&^	121.611	<0.001
Glu (mmol/L)	8.1±1.0	15.7±1.3**	4.2±0.7^##^	6.5±0.6^##^^,^ ^&&^	188.908	<0.001
IL-1β(pg/mL)	118.6±22.2	316.7±30.1**	238.3±42.0^##^	215.5±27.2^##^	54.507	<0.001
IL-18(pg/mL)	47.2±12.5	85.9±7.0**	57.1±6.2^##^	55.5±5.2^##^	33.855	<0.001

** *p* < 0.01 vs. WT group

^##^ *p* < 0.01 vs. SED group

^&&^ *p* < 0.01 vs. EX-1h group.

### Effects of aerobic exercise on serum FGF21 level, and possible tissue sources of serum FGF21

**Fig 3:**
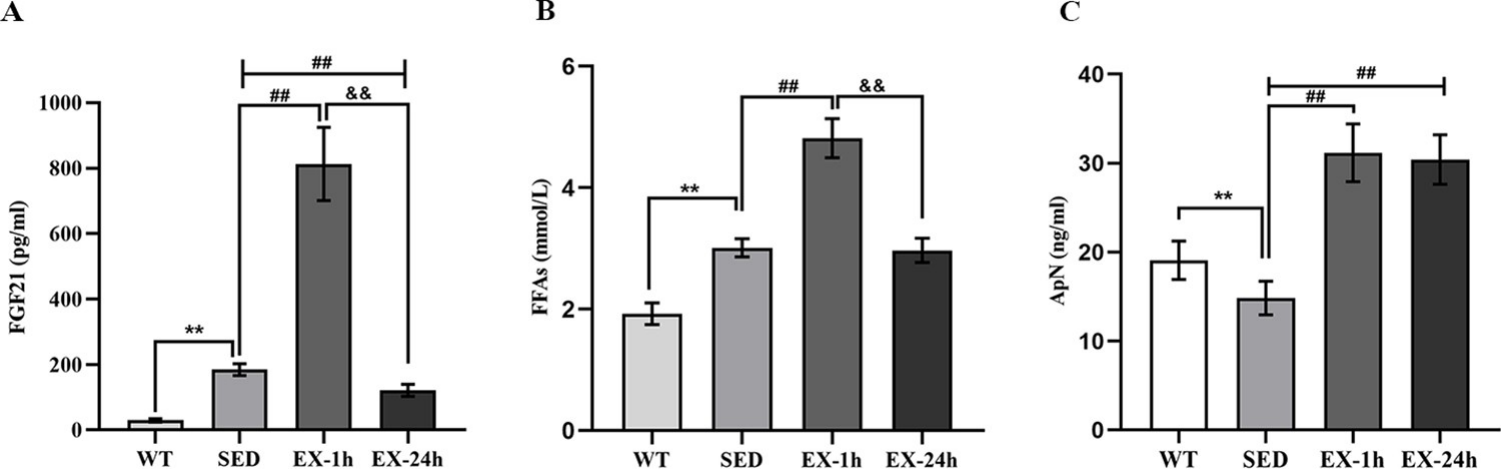
The effect of aerobic exercise on serum FGF21, FFAs, ApN levels. (**A**) Aerobic exercise affects serum FGF21 levels. (**B**) Aerobic exercise affects serum FFAs levels. (**C**) Aerobic exercise affects serum ApN levels (n = 8). Data are expressed as mean ± SD. * *p* < 0.05, ** *p* < 0.01 vs. WT group; ^##^ *p* < 0.01 vs. SED group; ^&&^ *p* < 0.01 vs. EX-1h group.

We collected blood samples at 1 h and 24 h after exercise to determine the effect of aerobic exercise on serum FGF21 level and to observe the fluctuations in serum FGF21 level after exercise. The SED group had higher serum FGF21 level than the WT controls (approximately 6-fold, *p* < 0.01, 184.3 ± 18.0 pg/mL vs. 29.6 ± 5.3 pg/mL). The serum FGF21 level was higher in the EX-1h group than in the SED group (approximately 5-fold, *p* < 0.01, 813.4 ± 111.8 pg/mL vs. 184.3 ± 18.0 pg/mL). The serum FGF21 level in the EX-24h group was significantly lower than that in the EX-1h and SED groups (*p* < 0.01, 121.4 ± 18.2 pg/mL vs. 813.4 ± 111.8 pg/mL and 184.3 ± 18.0 pg/mL; Fig 3A).

The serum FFA and adiponectin (ApN) levels were examined to explore the possible mechanisms by which aerobic exercise modulates serum FGF21 level. The results showed that the serum FFA levels in the SED group were higher than those in the WT group (*p* < 0.01); the level of serum FFAs in the EX-1h group was higher than that in the SED group (*p* < 0.01). The EX-24h group presented lower serum FFA levels than the EX-1h group (*p* < 0.01), but there was no significant difference from those of the SED group (p > 0.05; Fig 3B). The serum ApN level in the SED group was lower than that in the WT group (*p* < 0.01), and the serum adiponectin level in the EX-1h and EX-24h groups was higher than that in the SED group (*p* < 0.01). There was no significant difference between the EX-1h and EX-24h groups (*p* > 0.05; Fig 3C).

**Fig 4:**
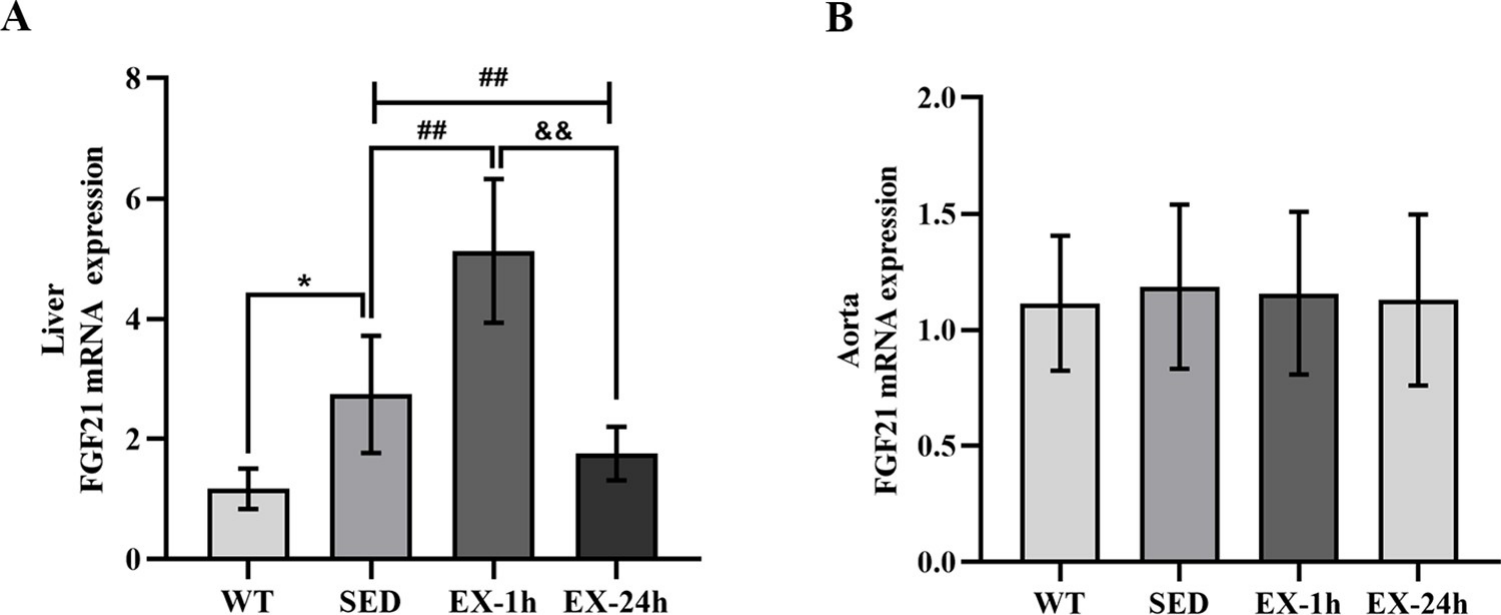
The effect of aerobic exercise on the expression of liver and aorta FGF21 mRNA. (**A**) Aerobic exercise affects FGF21 mRNA expression in liver; (**B**) Aerobic exercise did not alter the expression of FGF21 mRNA in the aorta (n = 7). Data are expressed as mean ± SD of three experiments. * *p* < 0.05, ** *p* < 0.01 vs. WT group; ^##^ *p* < 0.01 vs. SED group; ^&&^ *p* < 0.01 vs. EX-1h group.

**Fig 5:**
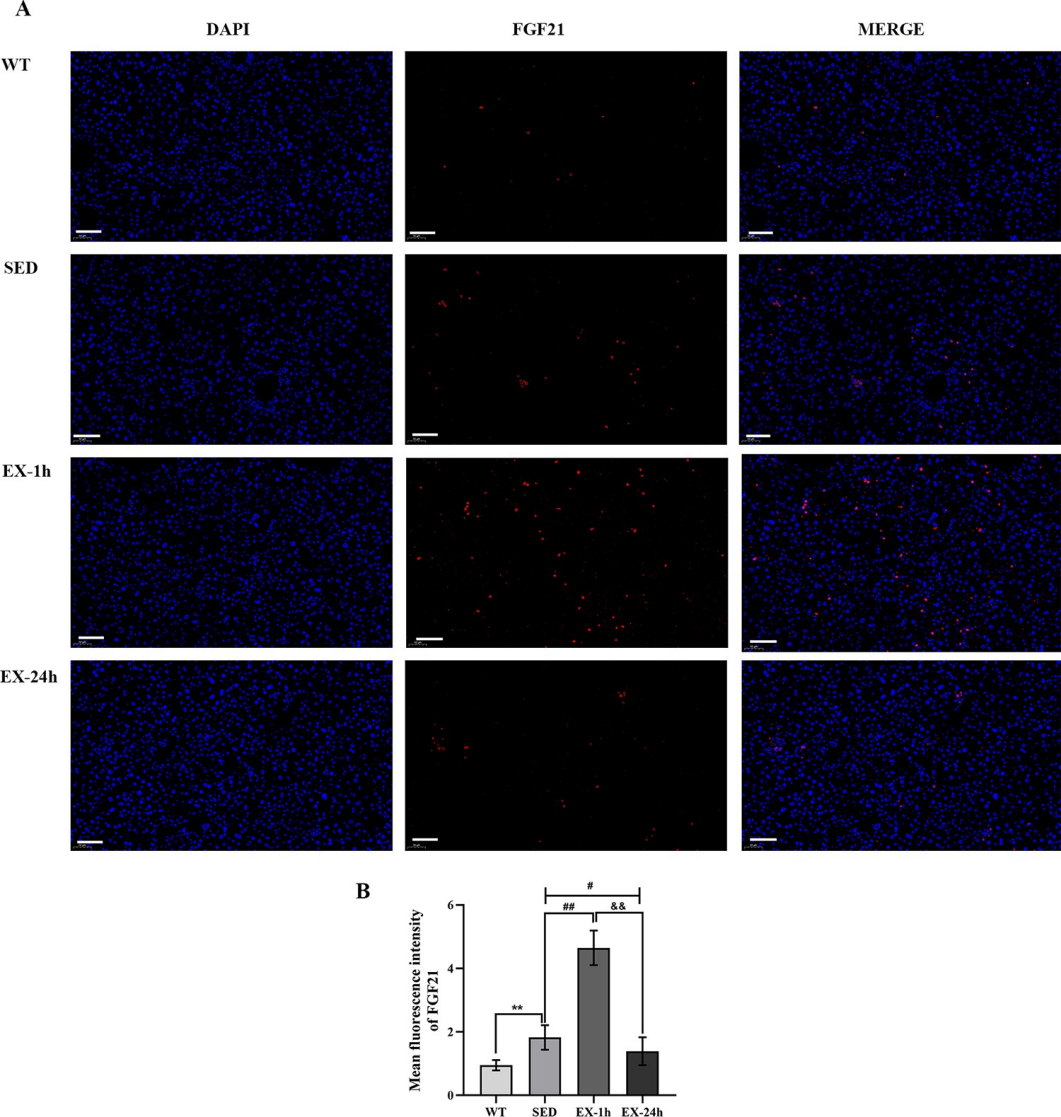
Aerobic exercise activates liver FGF21 protein expression. (**A**) Immunofluorescence detects liver FGF21 protein expression level. Scale bar = 100 μm. (**B**) Mean fluorescence intensity analysis of FGF21 in liver. The data are expressed as mean ± SD (n = 5, three slices taken from each mouse for staining), ** *p* < 0.01 vs. WT group; ^##^ *p* < 0.01 vs. SED group; ^&&^ *p* < 0.01 vs. EX-1h group.

**Fig 6:**
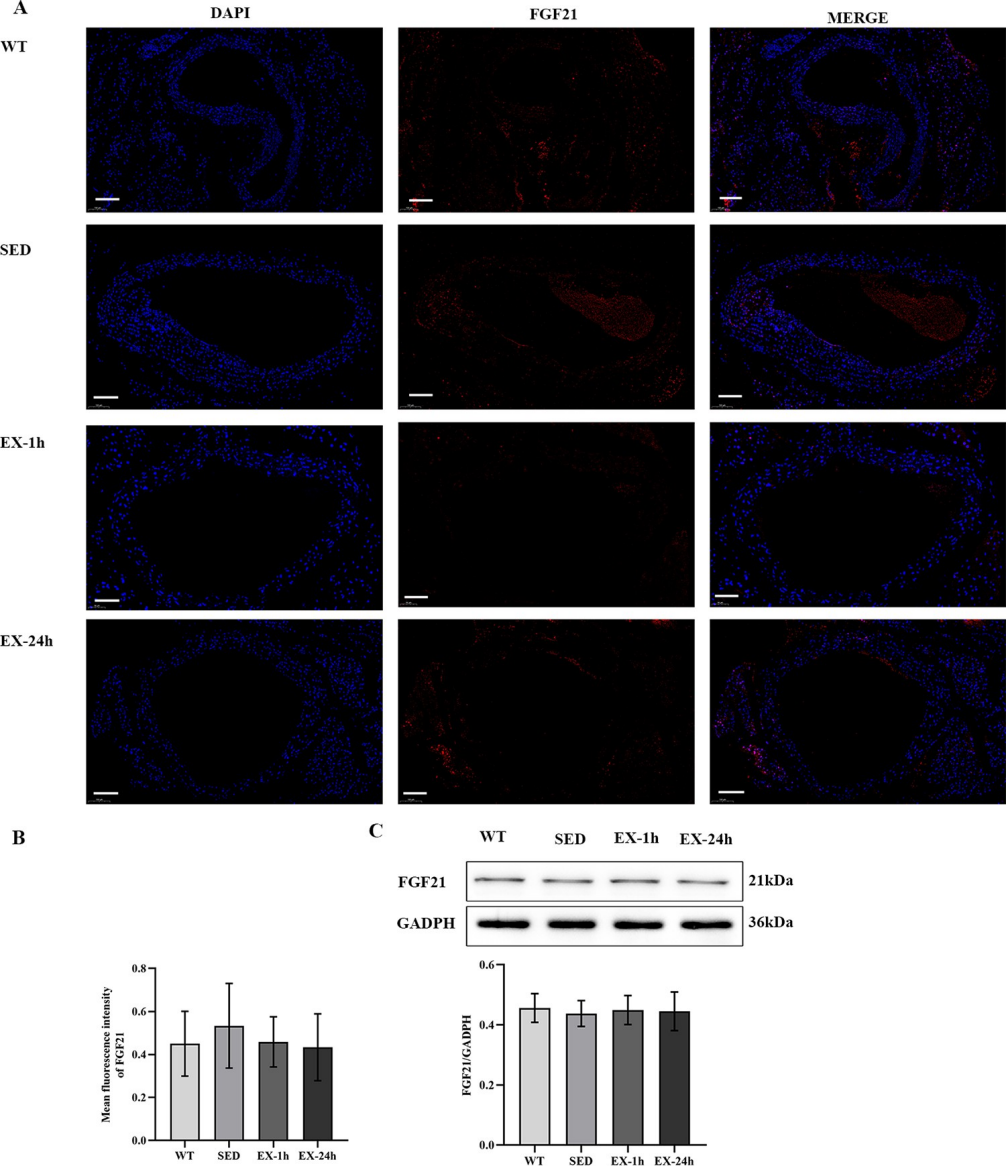
Aerobic exercise does not change the expression of FGF21 protein in the aorta. (**A**) The expression level of FGF21 protein in the aorta was detected by immunofluorescence. Scale bar = 100 μm. (**B**) Analysis of the average fluorescence intensity of FGF21 in the aorta (n = 5, three slices taken from each mouse for staining). (**C**) The protein levels of FGF21 in the aorta were detected by western blotting (n = 7). Data are presented as mean ± SD of three experiments.

RT-PCR, western blotting, and immunofluorescence assays were used to detect FGF21 expression in the liver and aorta to verify the tissue origin of FGF21 during aerobic exercise. The FGF21 mRNA and protein levels in the liver of the SED group were significantly higher than those of the WT group (*p* < 0.05); the FGF21 mRNA and protein levels of the EX-1h group were higher than those of the SED group (*p* < 0.01). The FGF21 mRNA and protein levels of the EX-24h group were lower than those of the SED and EX-1h groups (*p* < 0.01; Fig 4A, and Fig 5A and 5B). FGF21 mRNA and protein expression in the aortas did not differ among the four groups (Fig 4B, and Fig 6A–6C). These findings indicate that, during aerobic exercise, serum FGF21 may be produced by the liver rather than the aorta.

### Aerobic exercise inhibits aortic NLRP3 inflammasome-mediated pyroptosis

**Fig 7:**
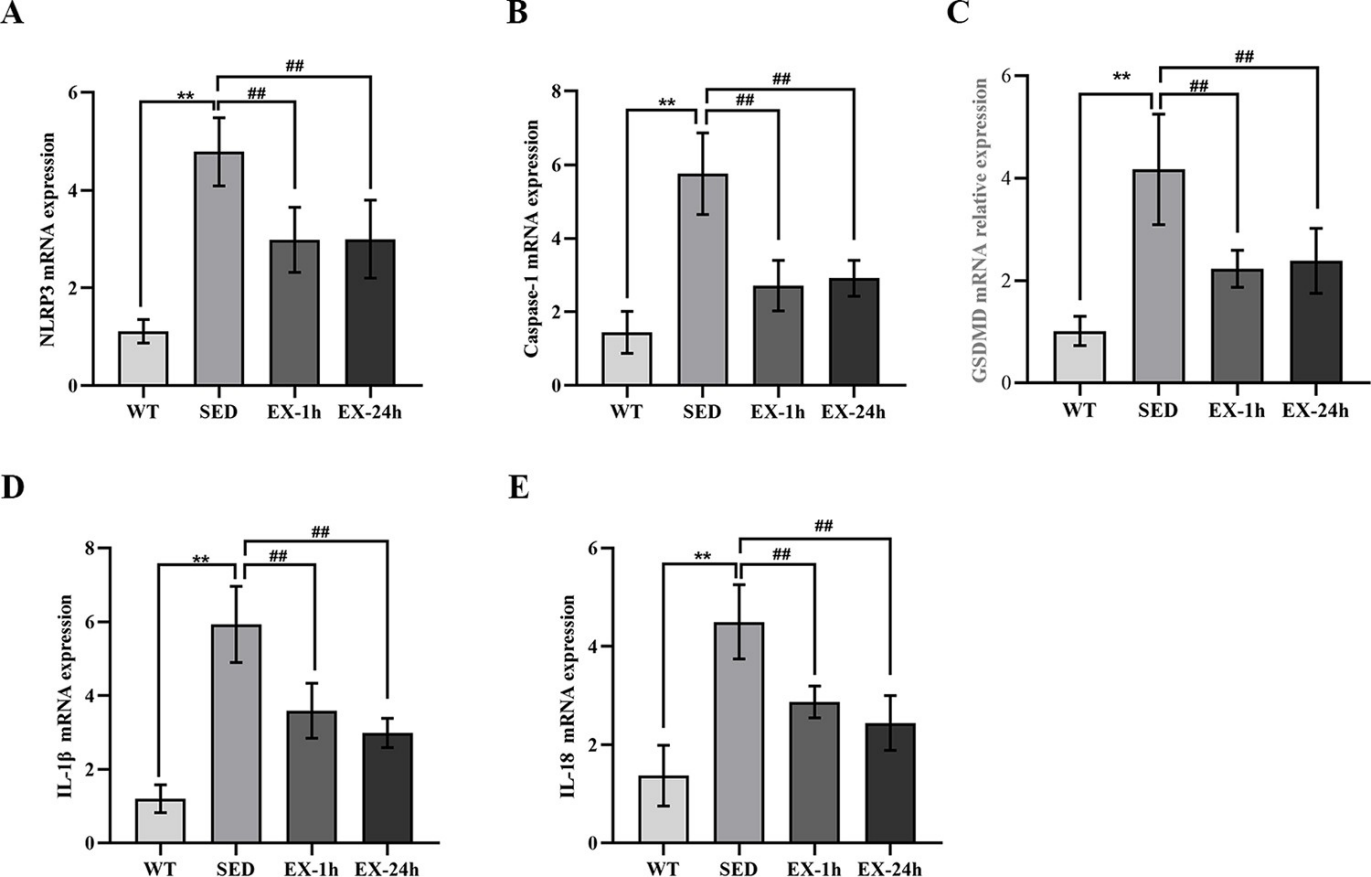
Aerobic exercise inhibits NLRP3 inflammasome-mediated pyroptosis-related factor mRNA expression in the aorta. (**A**-**E**) The mRNA expression of NLRP3, caspase-1, GSDMD, IL-1β, and IL-18 in the aorta were detected by real-time polymerase chain reaction (n = 7). Data are presented as mean ± SD of three experiments, **p < 0.01 vs. WT group; ^##^*p* < 0.01 vs. SED group.

**Fig 8:**
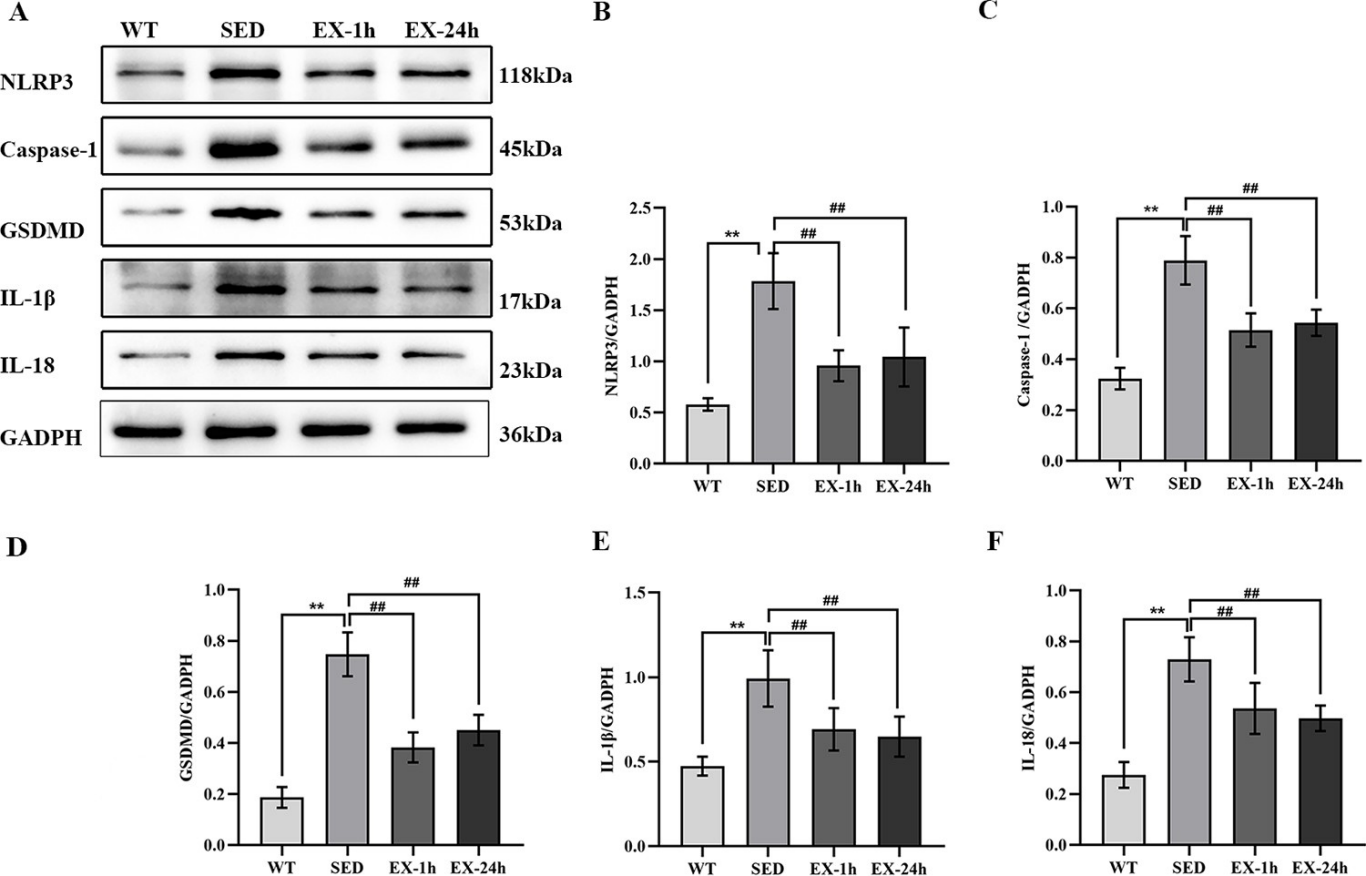
Aerobic exercise inhibits NLRP3 inflammasome-mediated pyroptosis-related factor protein expression in the aorta. (**A**) The protein expression of NLRP3, caspase-1, GSDMD, IL-1β, and IL-18 in the aorta were detected by western blotting. (**B**-**F**) Comparison of protein expression in each group (n = 7). Data are presented as mean ± SD of three experiments, ** *p* < 0.01 vs. WT group; ^##^ *p* < 0.01 vs. SED group.

**Fig 9:**
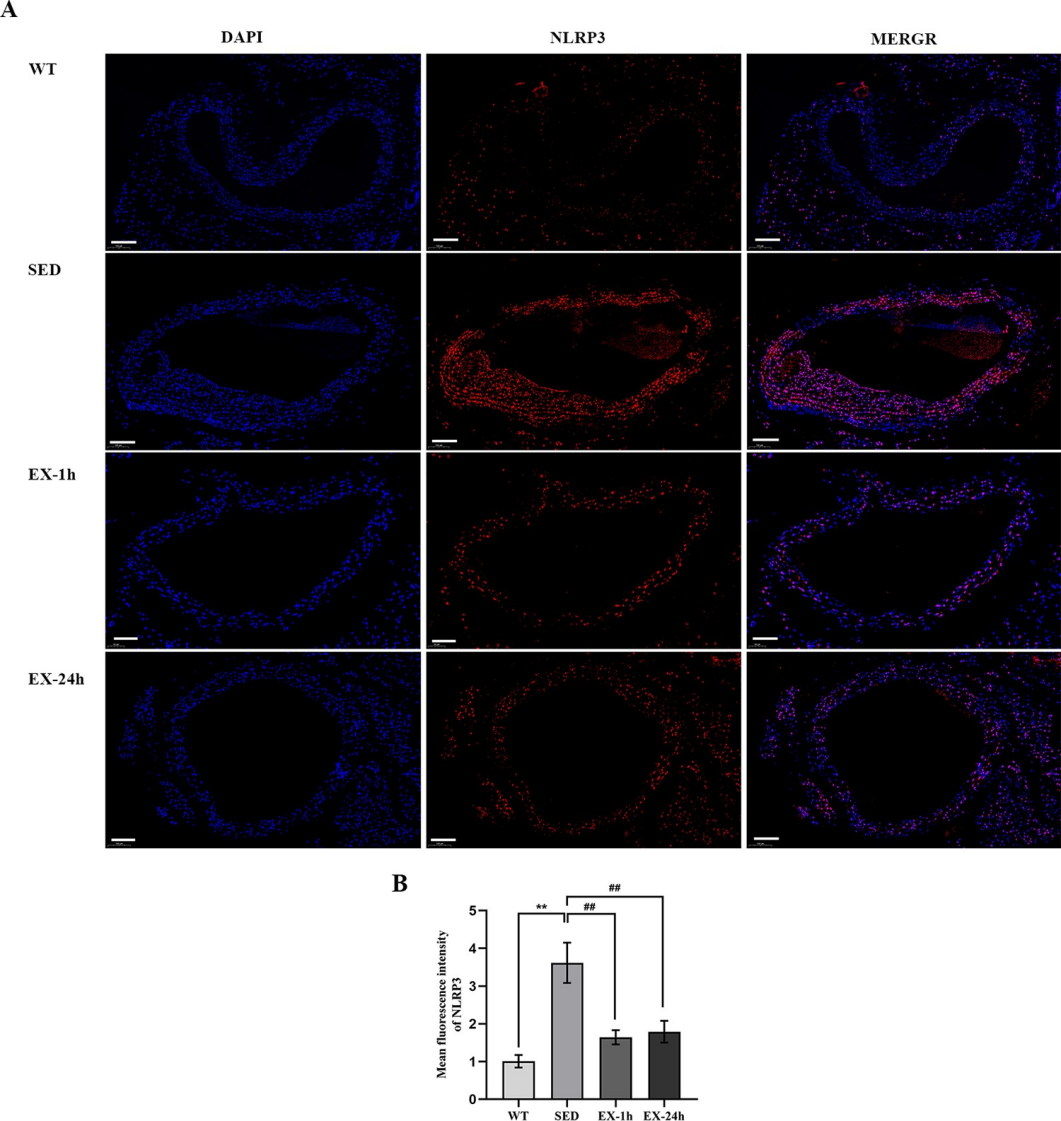
Aerobic exercise inhibits the expression of NLRP3 protein in the aorta. (**A**) The expression of NLRP3 protein in the aorta was detected by the immunofluorescence method. Scale bar = 100 μm. (**B**) Analysis of the average fluorescence intensity of NLRP3 in the aorta (n = 5, three slices taken from each mouse for staining). The data are expressed as mean ± SD. ** *p* < 0.01 vs. WT group; ^##^ *p* < 0.01 vs. SED group.

We used RT-PCR to measure mRNA levels of genes encoding the pyroptosis-related markers NLRP3, caspase-1, GSDMD, IL-1β, and IL-18 in the aorta; we measured protein expression levels using western blotting. Meanwhile, we used immunofluorescence to detect aortic NLRP3. We found that both mRNA and protein expression levels of all five pyroptosis markers in the SED group were significantly higher than those in the WT group (*p* < 0.01). The mRNA and protein expression of the above markers in the EX-1h and EX-24h groups were significantly lower than those in the SED group (*p* < 0.01). No significant differences were observed between the EX-1h and EX-24h groups (Fig 7A–7E, Fig 8A–8F, and Fig 9A and 9B).

## Discussion

Our study revealed the following. First, aerobic exercise increased the sensitivity of FGF21 by upregulating the expression of the downstream receptor ApN; the serum FGF21 level after exercise increased initially, and then decreased. Second, aerobic exercise downregulated the expression of NLRP3 inflammasome-mediated pyroptosis-related markers in the aorta, and FGF21 may participate in the above process. Third, the liver may be the tissue source of serum FGF21 during aerobic exercise. These findings provide new information on the molecular mechanisms by which aerobic exercise inhibits atherosclerosis.

Aerobic exercise can reduce body weight and blood glucose and lipid levels, and inhibit the formation of atherosclerosis, which has been confirmed by previous studies [37, 38, 40]. Our results are consistent with those of previous studies. That is, the body weight, blood glucose and lipid levels, and proportion of plaque area of mice in the EX-1h and EX-24h groups were lower than those of mice in the SED group. Notably, some studies have reported that aerobic exercise does not alter body weight, and blood glucose and lipid levels [39, 41, 42]. This may be related to the different patterns, frequencies, intensities, and durations of exercise in different studies. Therefore, researchers should take these into account when formulating exercise programs.

During recent years, multiple studies have shown that pyroptosis is an important pathogenesis of atherosclerosis [7, 11]. We established a mouse model of atherosclerosis by feeding *ApoE*^*-/-*^ mice a high-fat diet. The expression of pyroptosis-related markers was upregulated in the aorta of sedentary atherosclerotic mice (SED group), and this is consistent with the finding of previous studies [7, 11]. The NLRP3 inflammasome is an important driver of atherosclerosis [43]. Risk factors, such as oxidative stress, hyperglycemia, dyslipidemia, inflammation, mitochondrial dysfunction, and endoplasmic reticulum stress can activate the NLRP3 inflammasome [44–46]. Inhibition or reversal of these risk factors should eliminate the overactivated NLRP3 inflammasome and ultimately inhibit pyroptosis. Studies have shown that exercise can reduce cardiac oxidative stress and NLRP3 inflammasome expression in *ApoE*^*-/-*^ mice [37]. Lee et al. [47] found that exercise training could reduce the upregulated expression of NLRP3 and its downstream cascade factors in an obese mouse model. In a diabetic mouse model, exercise restored endothelial nitric oxide synthase (eNOS) expression and nitric oxide (NO) production by reducing NLRP3 expression, thereby eliminating downstream products of NLRP3 activation [48]. However, it is not clear whether aerobic exercise can inhibit NLRP3 inflammasome-mediated pyroptosis in the aorta. Our results showed that 12 weeks of aerobic exercise downregulated the mRNA and protein levels of NLRP3, caspase-1, GSDMD, IL-1β, and IL-18 in the aorta, indicating that aerobic exercise can inhibit NLRP3 inflammasome-mediated pyroptosis in the aorta.

FGF21 was first discovered in mouse embryos in 2000 [49]. It has various metabolic benefits [22, 50]. Paradoxically, patients with atherosclerosis have high serum FGF21 levels [16, 26]. FGF21 is an independent predictor of and risk marker for atherosclerosis [51]. We found that high fat consumption elevated serum FGF21 level in *ApoE*^*-/-*^ mice compared with that in normal diet-fed C57BL/6J controls. Previous studies have shown that mice fed a high-fat diet, and patients and animals with obesity or diabetes have higher levels of endogenous FGF21, but its physiological function is considerably lower than that of exogenous recombinant FGF21 [52, 53]. In other words, a high concentration does not indicate optimal function. ApN is produced by fat cells and is an effector of FGF21 [29, 54]. ApN deficiency disrupts glucose and lipid regulation by FGF21 [54]. Moreover, ApN levels in patients with atherosclerosis are considerably reduced [29]. FGF21 requires FGF receptor-1 (FGFR1) and the co-receptor β-Klotho (KLB) on the cell surface to initiate the intracellular signaling cascade that produces its physiological effects. However, high levels of TNFα and IL-1β in patients with atherosclerosis have been shown to decrease the expression of KLB [25, 28]. This shows that in metabolic diseases such as atherosclerosis, the downstream signaling pathway of FGF21 is disrupted and a state of FGF21 resistance appears, leading to an increase in the concentration of FGF21 to compensate.

FGF21 is an exercise reactive factor in both rodents and humans [16]. It has been shown that serum FGF21 level was significantly increased in 14 healthy subjects who cycled for 45 min [35]. Campderrós et al. [55] measured serum FGF21 in 18 male runners before and after a marathon and found that at the end of the race, the FGF21 level was 20 times higher than that before the race. However, the serum FGF21 level reduced after 36 and 12 weeks of exercise in humans and animals with obesity [33, 34]. Therefore, the regulation of FGF21 by exercise is still controversial. Kim et al. [17] studied the trend of serum FGF21 level after 30 min of aerobic exercise in healthy adults, and found that its level peaked 1 h after exercise and returned to the pre-exercise level 2–3 h later. He et al. [35] observed that the serum FGF21 level peaked 1 h after exercise in 14 healthy individuals after cycling for 45 min. Our results show that the serum FGF21 level of EX-1h group is significantly higher than that of SED group. We further examined serum FFAs levels to explore the reasons for the rapid increase in the FGF21 level 1 h after exercise. The results showed that the level of serum FFAs in the EX-1h group was significantly higher than that in the SED group. Peroxisome proliferator-activated receptor-α (PPARα) is an upstream regulatory factor for FGF21, whose activity is stimulated by FFAs. During exercise, energy expenditure and lipolysis increase substantially in a short time, leading to a considerable increase in the serum FFA levels, which rapidly activates PPARα and upregulates FGF21 expression [56]. After exercise stimulation, PPARα expression returned to pre-exercise levels, and the serum FGF21 level was no longer elevated. Therefore, the serum FGF21 level after aerobic exercise may be affected by upstream regulatory factors, showing an increasing trend initially, and then decreasing.

Our study showed that the level of serum FGF21 at 24 h after exercise (the EX-24h group) was lower than that of the EX-1h and SED groups. Studies have shown that the serum FGF21 level in individuals or animals with obesity decreases after aerobic exercise [33, 34], consistent with the findings of our study. The expression of peroxisome proliferators activated receptor γ (PPARγ) was upregulated in adipocytes after aerobic exercise [57]. High expression of PPARγ can increase the secretion of ApN, a receptor downstream of FGF21, thereby increasing the activity of the FGF21- ApN axis [58]. Yang et al. [59] also found that 12 weeks of aerobic exercise could increase the serum ApN level in mice, thereby reducing the damage of the FGF21–ApN axis in diabetic mice. In this study, we observed the effect of aerobic exercise on the serum ApN level, and the results showed that the serum ApN level in the EX-1h and EX-24h groups was higher than that in the SED group. Thus, our results support that aerobic exercise increases the level of the downstream receptors of FGF21 (ApN), thereby enhancing FGF21 sensitivity. Studies have shown that FGF21 downstream receptors FGFR1, FGFR2, and KLB are also significantly upregulated after aerobic exercise, enhancing the physiological function of FGF21 [33, 56]. In conclusion, aerobic exercise upregulated the expression of downstream receptors of FGF21 and increased the sensitivity of FGF21. The increased sensitivity of FGF21 may regulate upstream target organs through a negative feedback, thereby reducing the secretion of FGF21. Therefore, the serum FGF21 level in the EX-24h group was lower than that in the SED group.

Studies have shown that there is a time effect of exercise on the regulation of FGF21 [17, 35]. Therefore, different blood collection times may lead to different serum FGF21 levels after exercise. The advantage of our study is that two time points, 1 and 24 h after exercise, were selected to detect changes in the FGF21 level. We also avoided bias in the results caused by diet, exercise style, and exercise intensity. We found that the serum FGF21 level rapidly increased 1 h after exercise, decreased 24 h after exercise, to levels even lower than that in the SED group, which explains the inconsistent effects of exercise on serum FGF21 observed in previous studies. Based on the above findings, we consider that each movement in a regular exercise is equivalent to supplementing a high dose of FGF21 to the body. However, the exercise increases the expression of the downstream receptor of FGF21, thereby increasing the sensitivity of FGF21, which is more meaningful in preventing atherosclerosis.

FGF21 has been shown to negatively regulate risk factors that activate the NLRP3 inflammasome [60–62]. Risk factors such as oxidative stress, hyperglycemia, dyslipidemia, inflammation, mitochondrial dysfunction, and endoplasmic reticulum stress can activate the NLRP3 inflammasome [51–53]. Mitochondrial dysfunction results in the excessive production of reactive oxygen species (ROS), which commonly activate the NLRP3 inflammasomes [63]. Endoplasmic reticulum stress induces mitochondrial Ca^2+^ overload and fragmentation, and increases permeability, triggering NLRP3 inflammasome-associated pyroptosis [64]. FGF21 has been shown to ameliorate ox-LDL-induced abnormal mitochondrial membrane potential, improving mitochondrial function and inhibit ox-LDL-induced endoplasmic reticulum stress, thereby reducing ROS production [24]. PGC-1α can enhance mitochondrial biosynthesis and function, and it is a downstream factor of FGF21. Exogenous FGF21 upregulated PGC-1α mRNA expression in mouse liver [65]. It also promoted cholesterol efflux and reduced cholesterol accumulation in foam cells [61, 62]. In previous studies, the advantages of FGF21 in reducing blood glucose, lipid levels, and energy balance have attracted considerable attention. However, research on its ability to restore mitochondrial function, ameliorate endoplasmic reticulum stress, and reduce cholesterol levels to prevent NLRP3 inflammasome activation has been limited. Our results showed that after 12 weeks of aerobic exercise, the levels of TG, TC, LDL-C, glucose, and inflammation in mice were decreased. The expression of pyroptosis-related markers (NLRP3, caspase-1, GSDMD, IL-1β, and IL-18) was also downregulated. These results are similar to the effect of exogenous use of FGF21 [24]. Our findings also revealed that the level of downstream receptors of FGF21 increased after aerobic exercise, thereby increasing the FGF21 sensitivity. Therefore, we speculate that activated FGF21 may be involved in aerobic exercise inhibiting NLRP3 inflammasome-mediated pyroptosis in the aorta, but further research is required.

The liver is the primary organ producing serum FGF21 [17]. FGF21 is also expressed in the aorta [18]; however, it is not clear whether aerobic exercise has effects on the liver, aorta, or other tissues to activate FGF21. In this study, the serum FGF21 level rapidly increased after acute exercise, and liver FGF21 expression increased significantly. However, there was no significant difference in aortic FGF21 expression among the four groups of mice. This indicates that the liver, but not the aorta, maybe a tissue source of FGF21 during aerobic exercise. The expression of hepatic FGF21 in the EX-24h group was lower than that in the SED group. Fletcher [33] also found that liver FGF21 mRNA and protein levels are decreased in obese mice after 36 weeks of exercise. This shows that FGF21 may also be subject to negative feedback after long-term exercise. When exercise intervention increases FGF21 sensitivity in circulation, a negative feedback may reduce hepatic production of FGF21. However, this is inconsistent with the results of some studies showing that the FGF21 level does not change after exercise [39, 66], indicating that the activation of FGF21 may also be affected by factors such as exercise intensity, exercise mode, and diet.

In summary, our study confirmed that aerobic exercise increased the sensitivity of FGF21. The serum FGF21 level after exercise increased initially, and then decreased. Aerobic exercise downregulated the expression of NLRP3 inflammasome-mediated pyroptosis-related markers in the aorta, and FGF21 may participate in the above process. Meanwhile, the liver may be the tissue source of serum FGF21 during aerobic exercise. However, there are some limitations to the study. First, there are different sources of FGF21 in the body. We only detected the expression of FGF21 in the liver and aorta, but the expression level of FGF21 in the adipose tissues, pancreas, muscle, and other tissues was not detected. Furthermore, we only analyzed the level of ApN; besides the FGF21–ApN signal axis, FGF21 may also combine with the FGFRs, KLB, and other downstream receptors to exert its physiological function. Finally, we did not inhibit the FGF21/NLRP3/pyroptosis pathway and thus could not elucidate the causal relationship between FGF21 and NLRP3 inflammasome-mediated pyroptosis during aerobic exercise. Researchers should consider the above limitations when referencing our research results. We will focus on resolving these limitations in our future studies.

## Supporting information

S1 Raw images (PDF)
