# Supplementary material for: Aerobic exercise regulates FGF21 and NLRP3 inflammasome-mediated pyroptosis and inhibits atherosclerosis in mice
Source: PLoS One. 2022 Aug 25;17(8):e0273527. doi: 10.1371/journal.pone.0273527 (PMC9409497; doi:10.1371/journal.pone.0273527)

Sample 1, Western blot 1, Figure 6C FGF21, 21 kDa

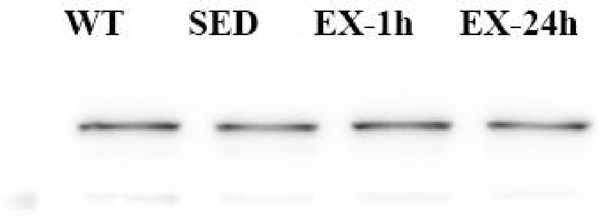

Sample 1, Western blot 1, Figure 6C GADPH , 36 kDa

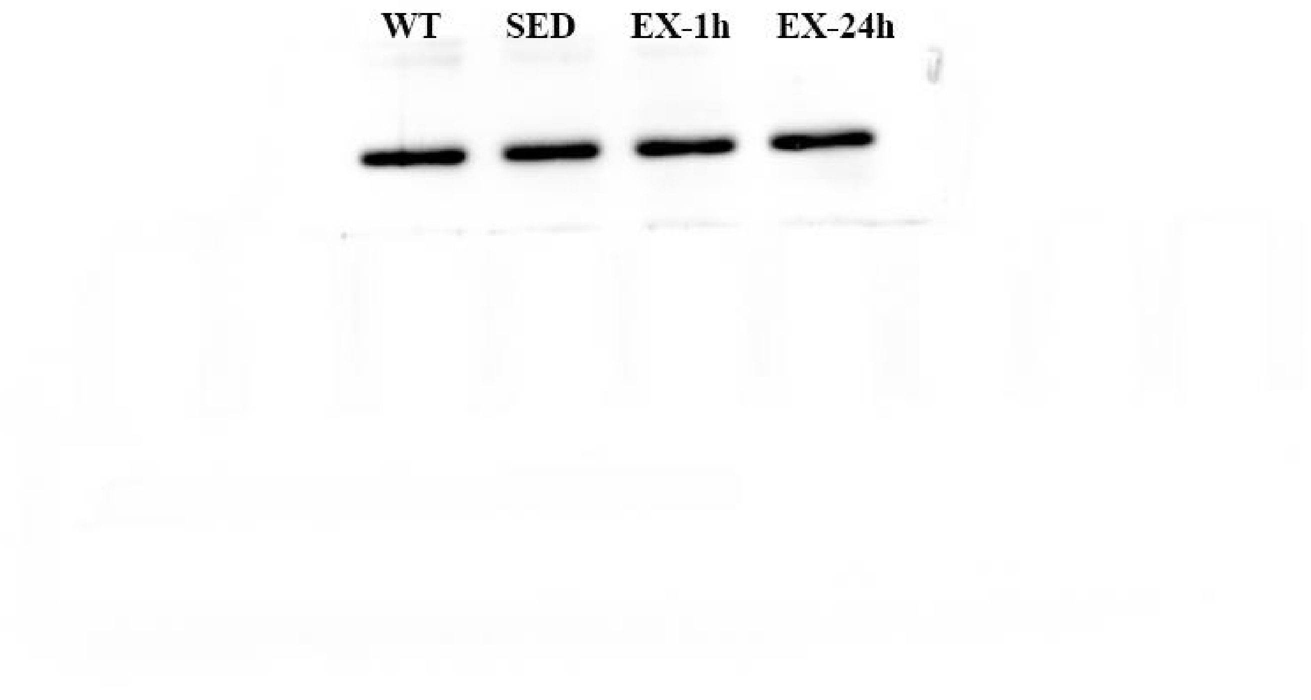

Sample 1, Western blot 2, FGF21, 21 kDa

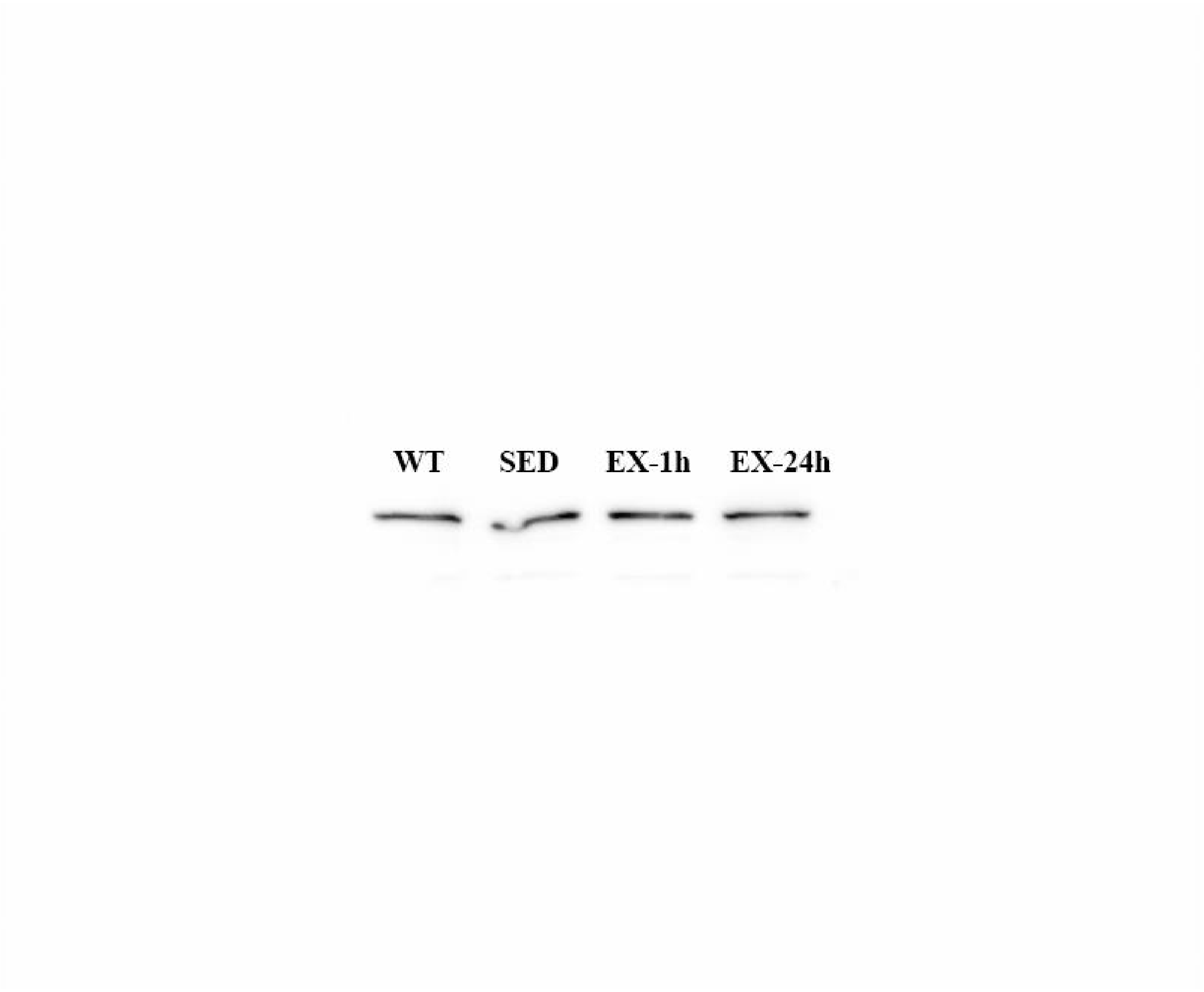

Sample 1, Western blot 2, GADPH, 36 kDa

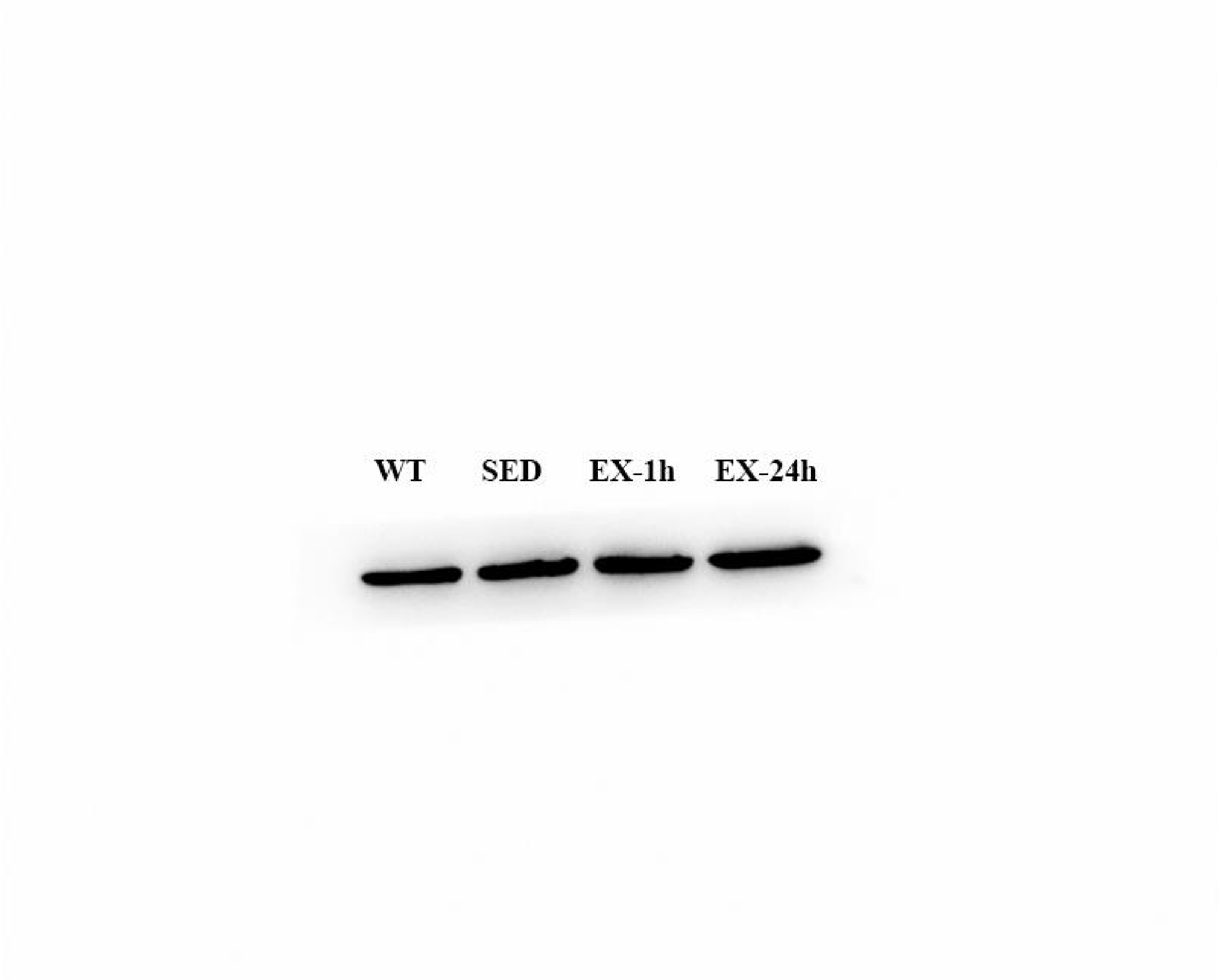

Sample 1, Western blot 3, FGF21, 21 kDa

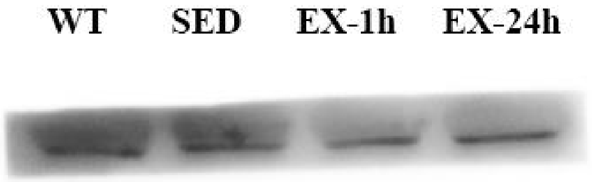

Sample 1, Western blot 3, GADPH, 36 kDa

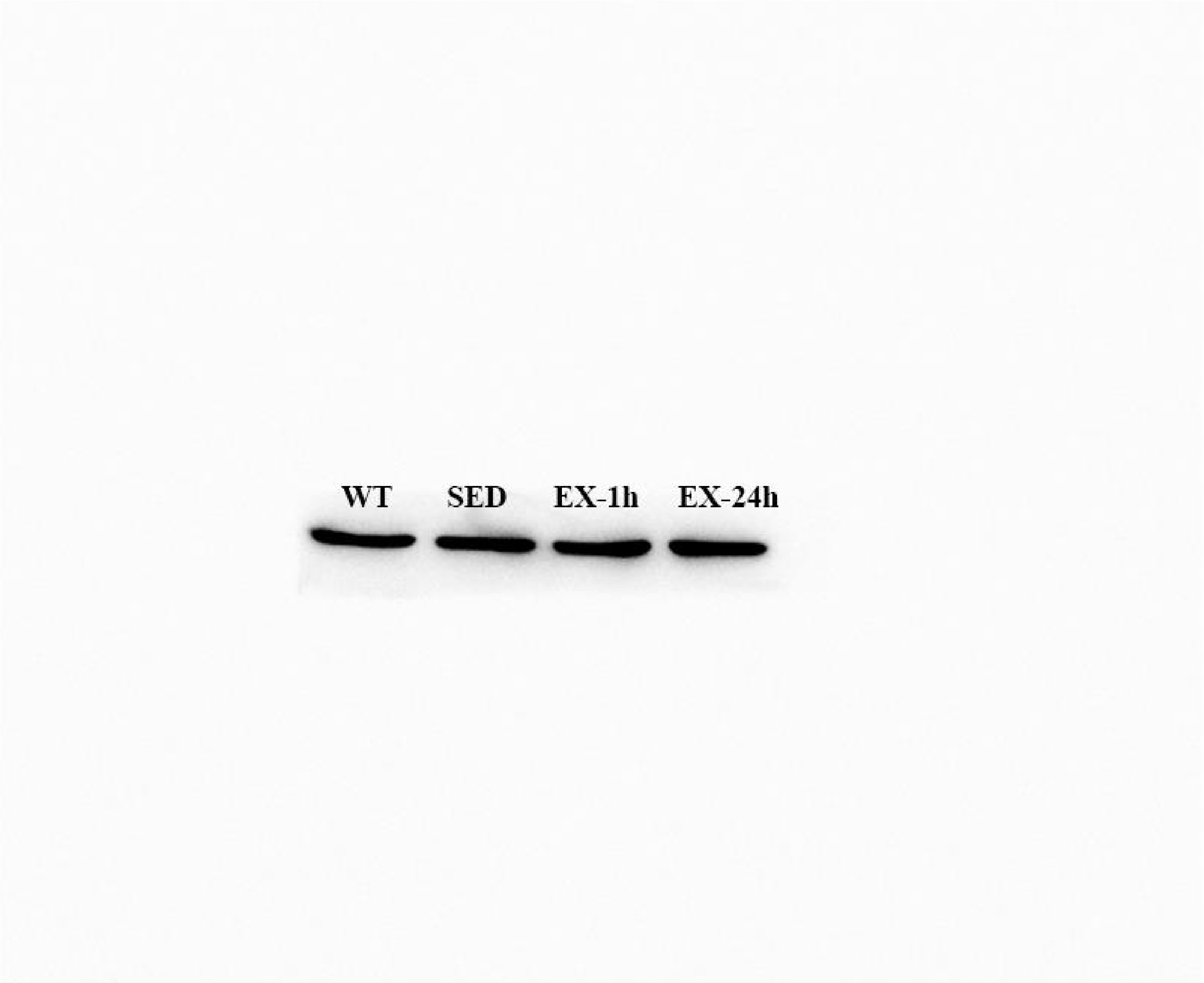

Sample 2, Western blot 1, Figure 8A NLRP3, 118 kDa

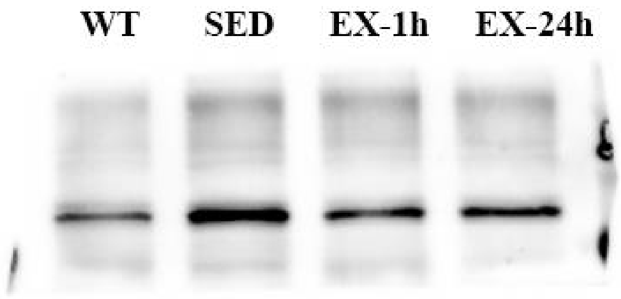

Sample 2, Western blot 1, Figure 8A caspase-1, 45 kDa

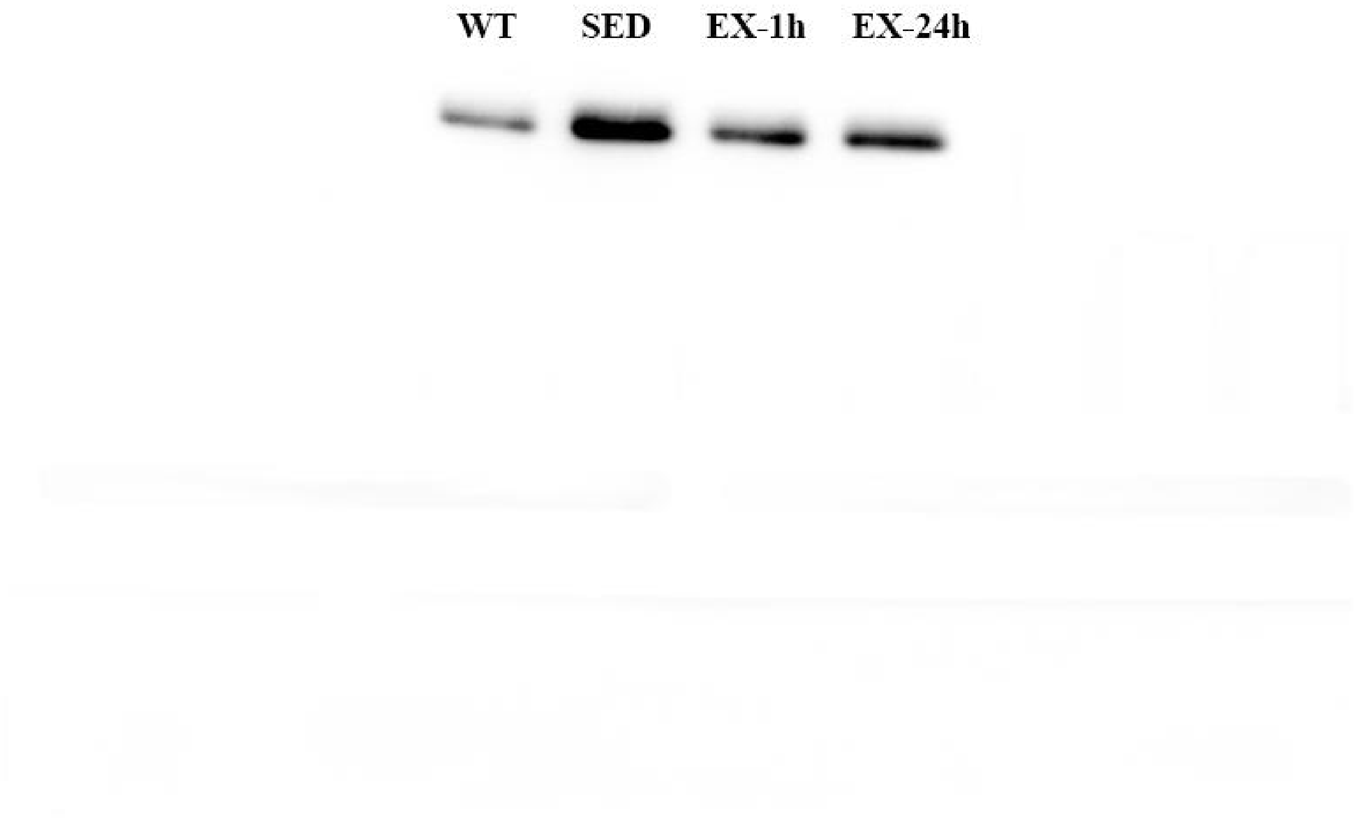

Sample 2, Western blot 1, Figure8A GSDMD, 53 kDa

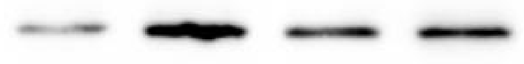

Sample 2, Western blot 1, Figure 8A IL-1β, 17 kDa

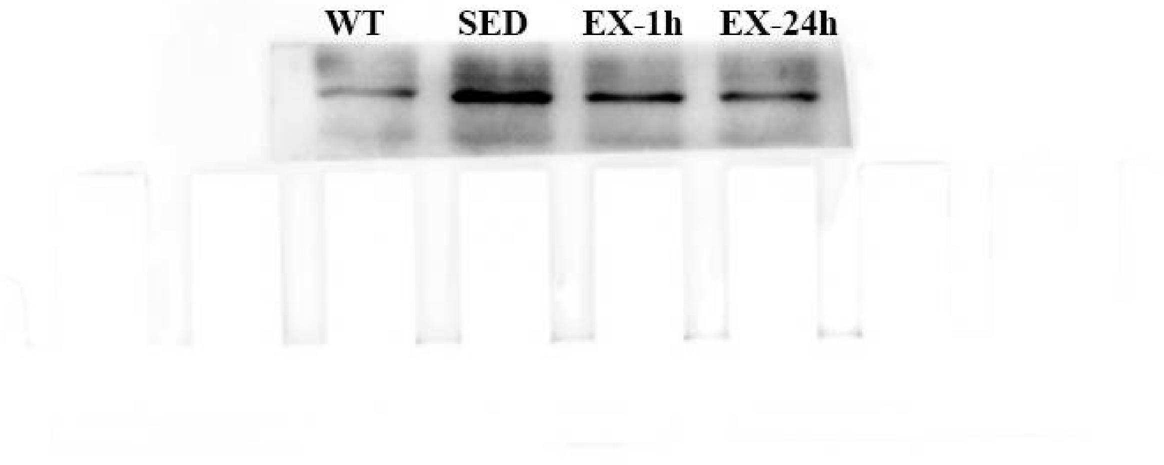

Sample 2, Western blot 1, Figure 8A IL-18, 23 kDa

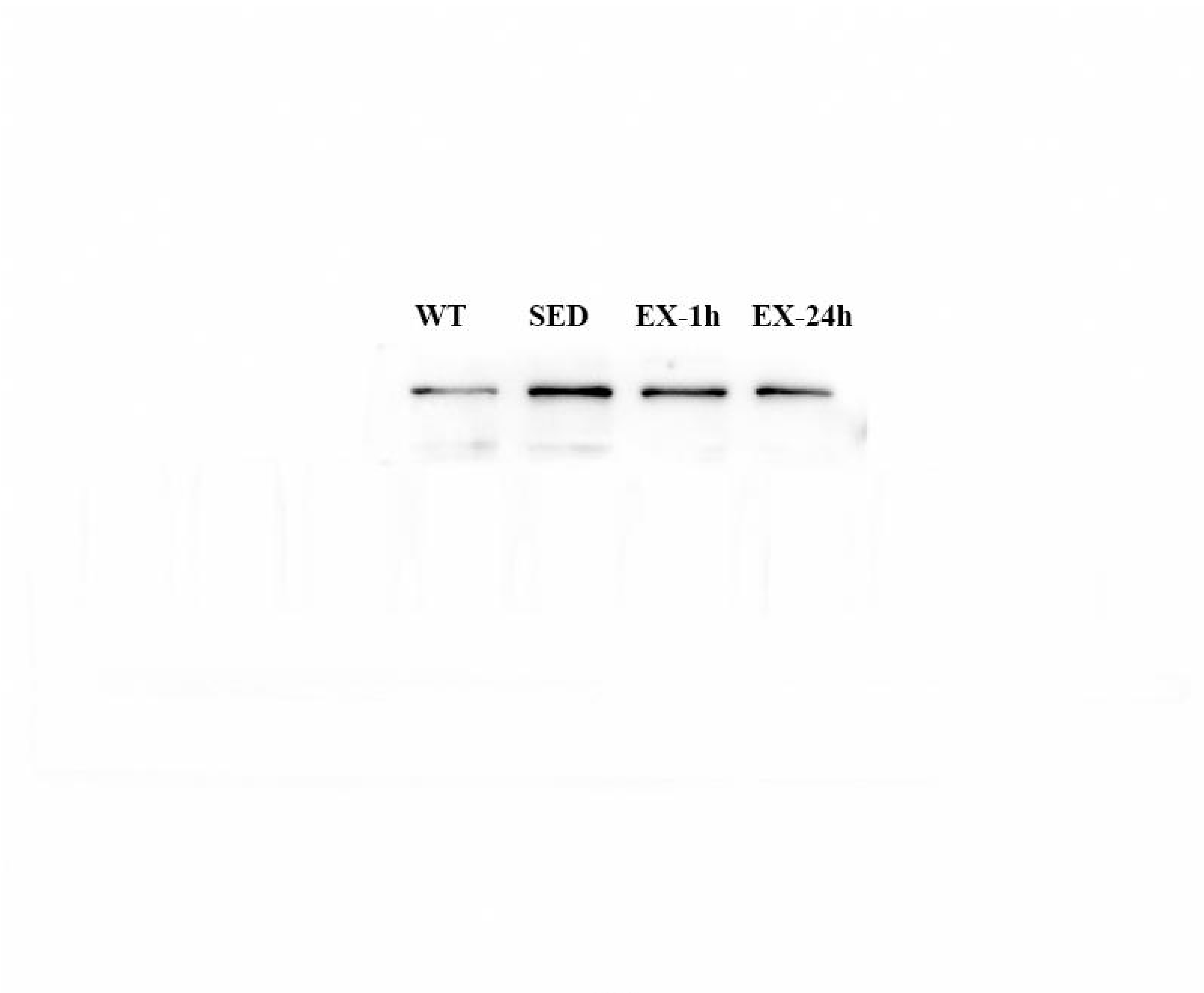

Sample 2, Western blot 1, Figure 8A GADPH, 36 kDa

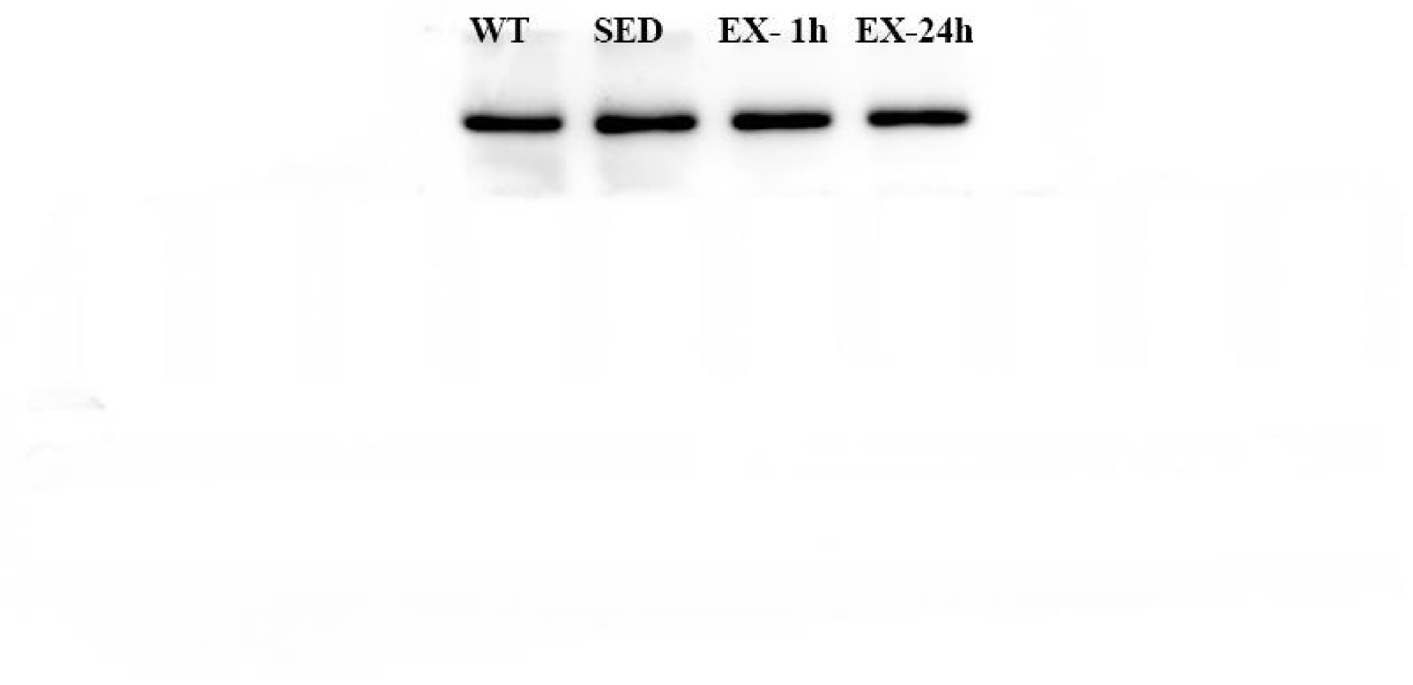

Sample 2, Western blot 2, NLRP3, 118 kDa

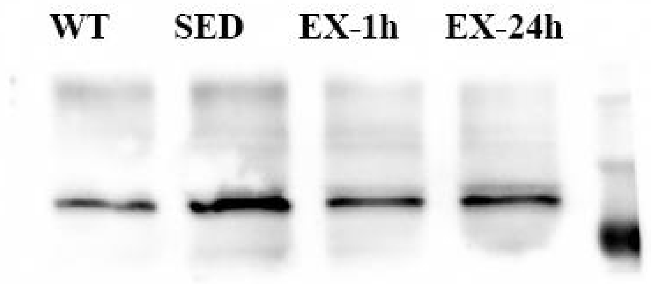

Sample 2, Western blot 2, caspase-1, 45 kDa

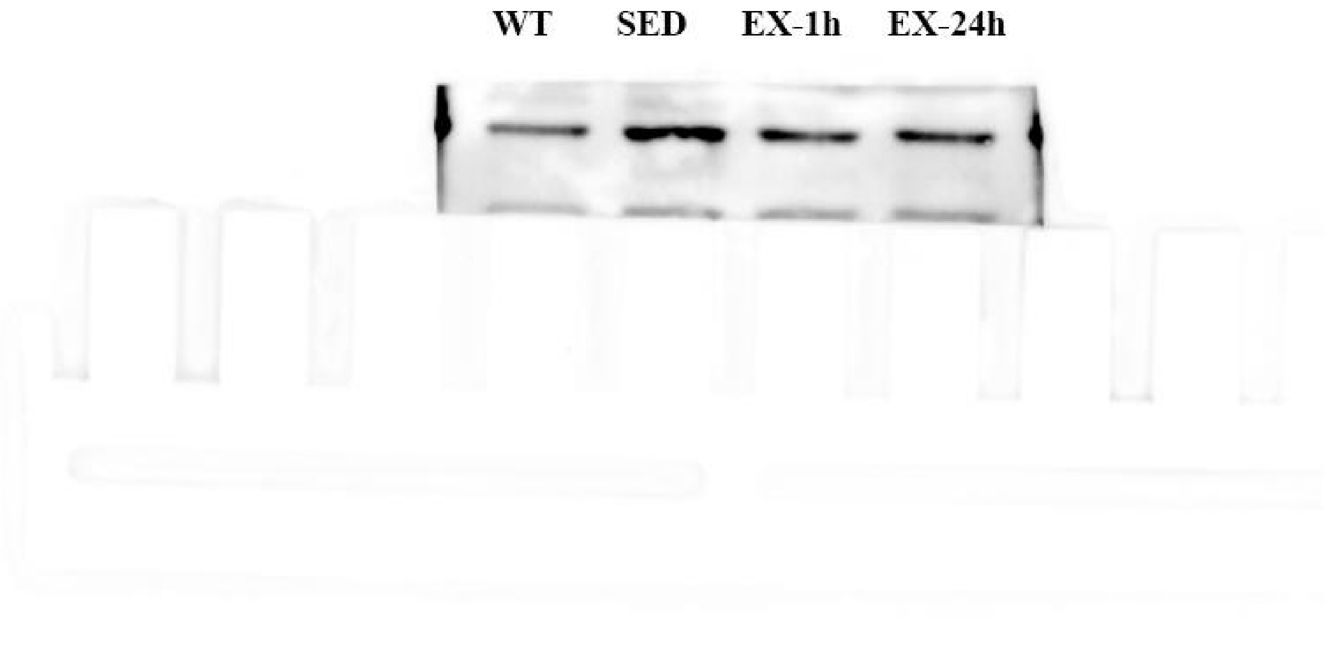

Sample 2, Western blot 2, GSDMD, 53 kDa

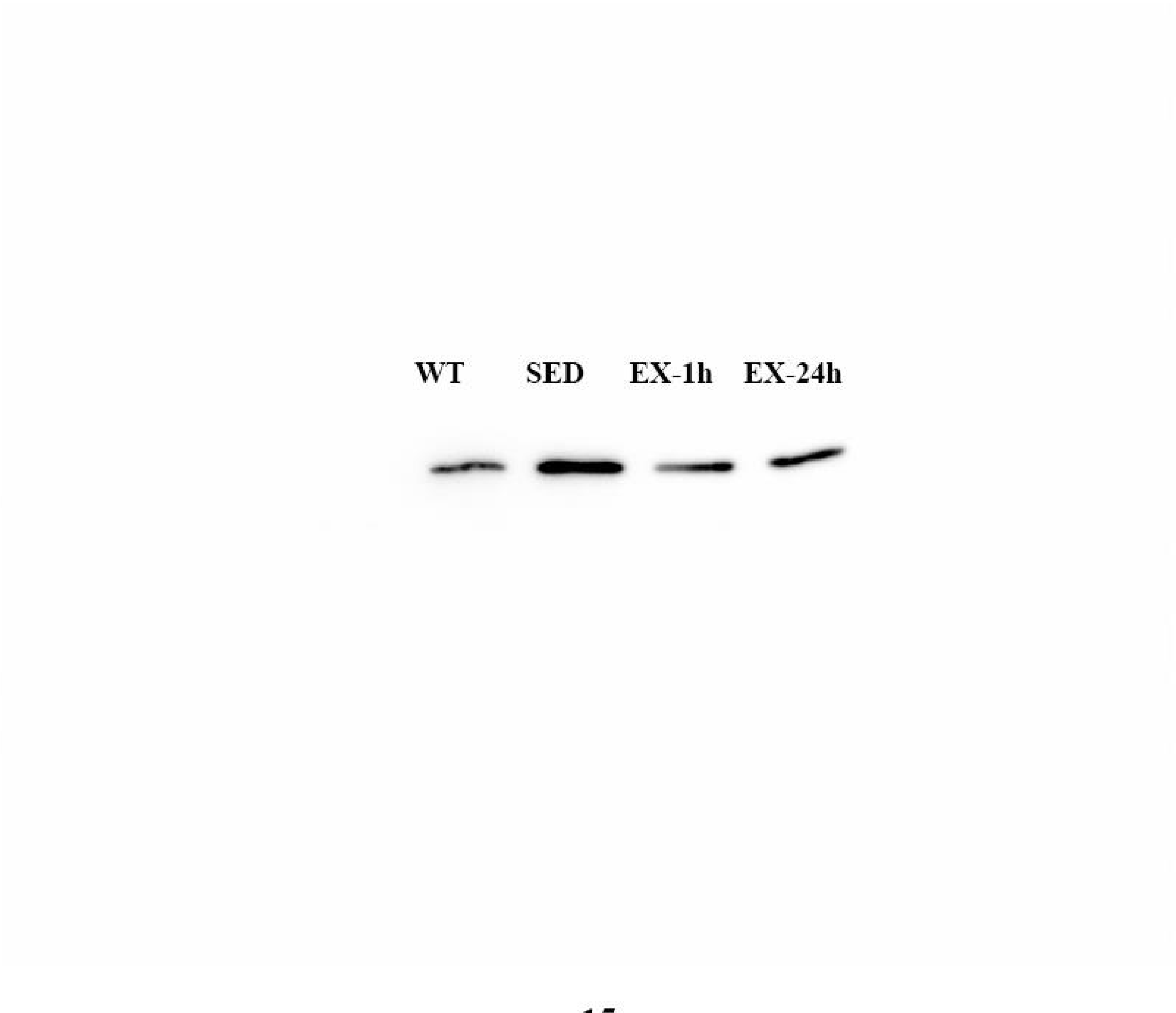

Sample 2, Western blot 2, IL-1β, 17 kDa

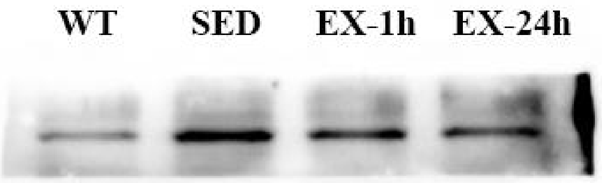

Sample 2, Western blot 2, IL-18, 23 kDa

WT SED EX-1h EX-24h

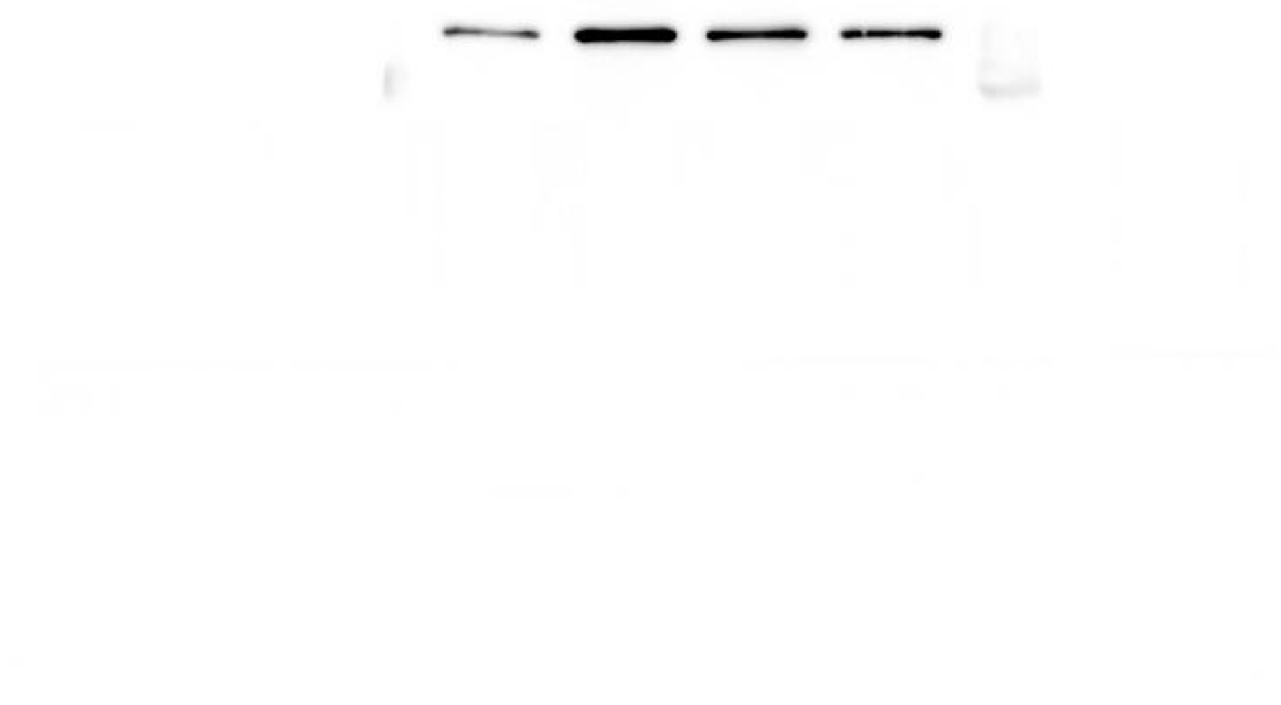

Sample 2, Western blot 2, GADPH, 36 kDa

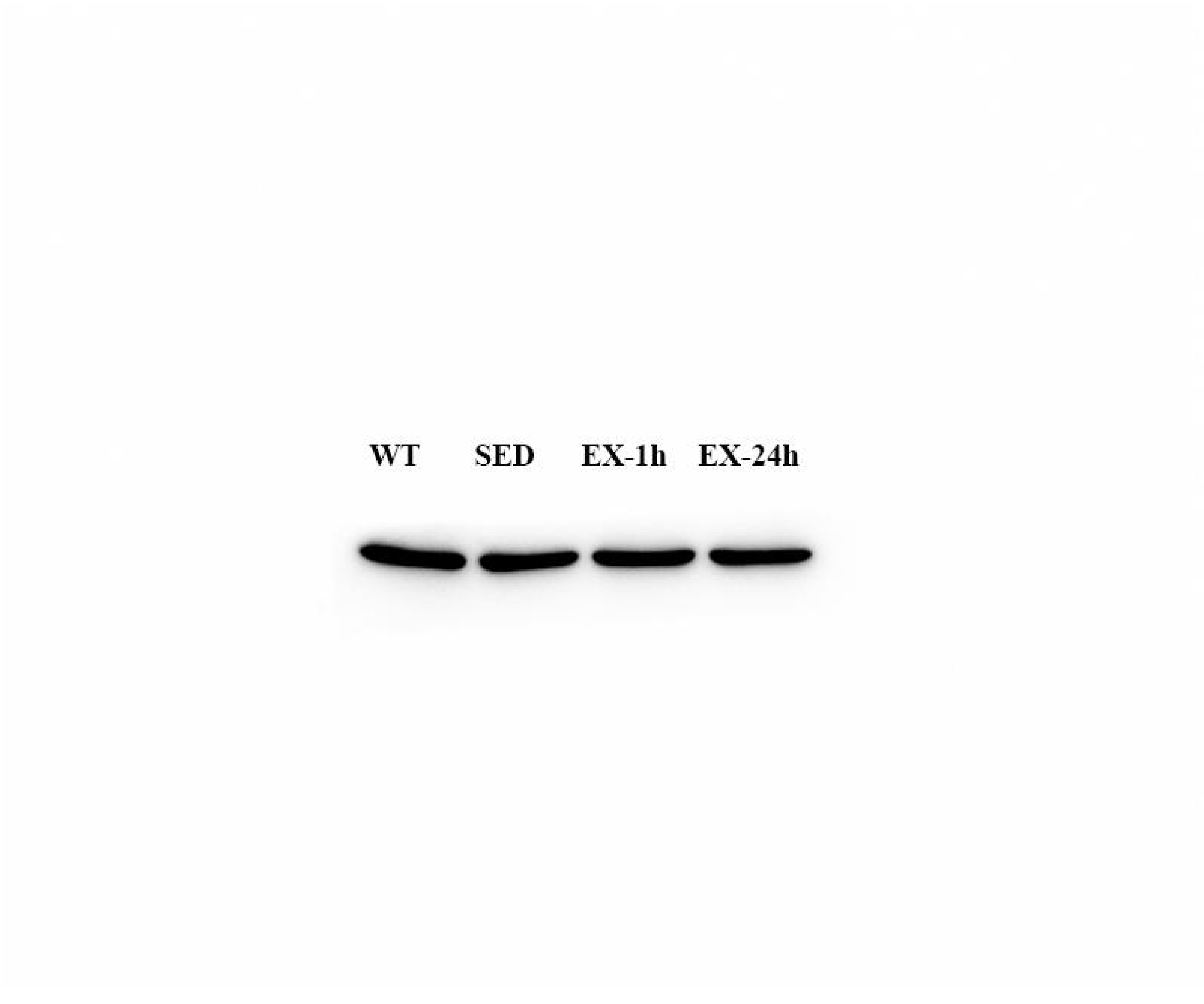

Sample 2, Western blot 3, NLRP3 , 118 kDa

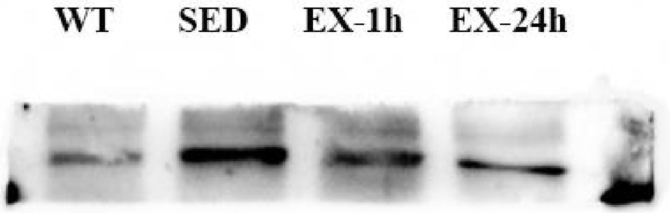

Sample 2, Western blot 3, caspase-1 , 45 kDa

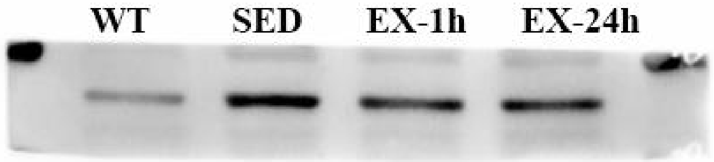

Sample 2, Western blot 3, GSDMD, 53 kDa

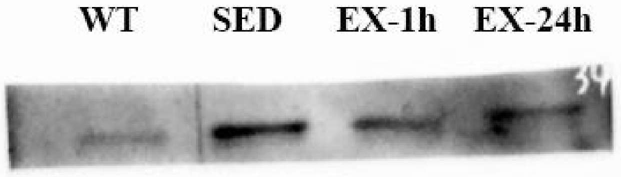

Sample 2, Western blot 3, IL-1β, 17 kDa

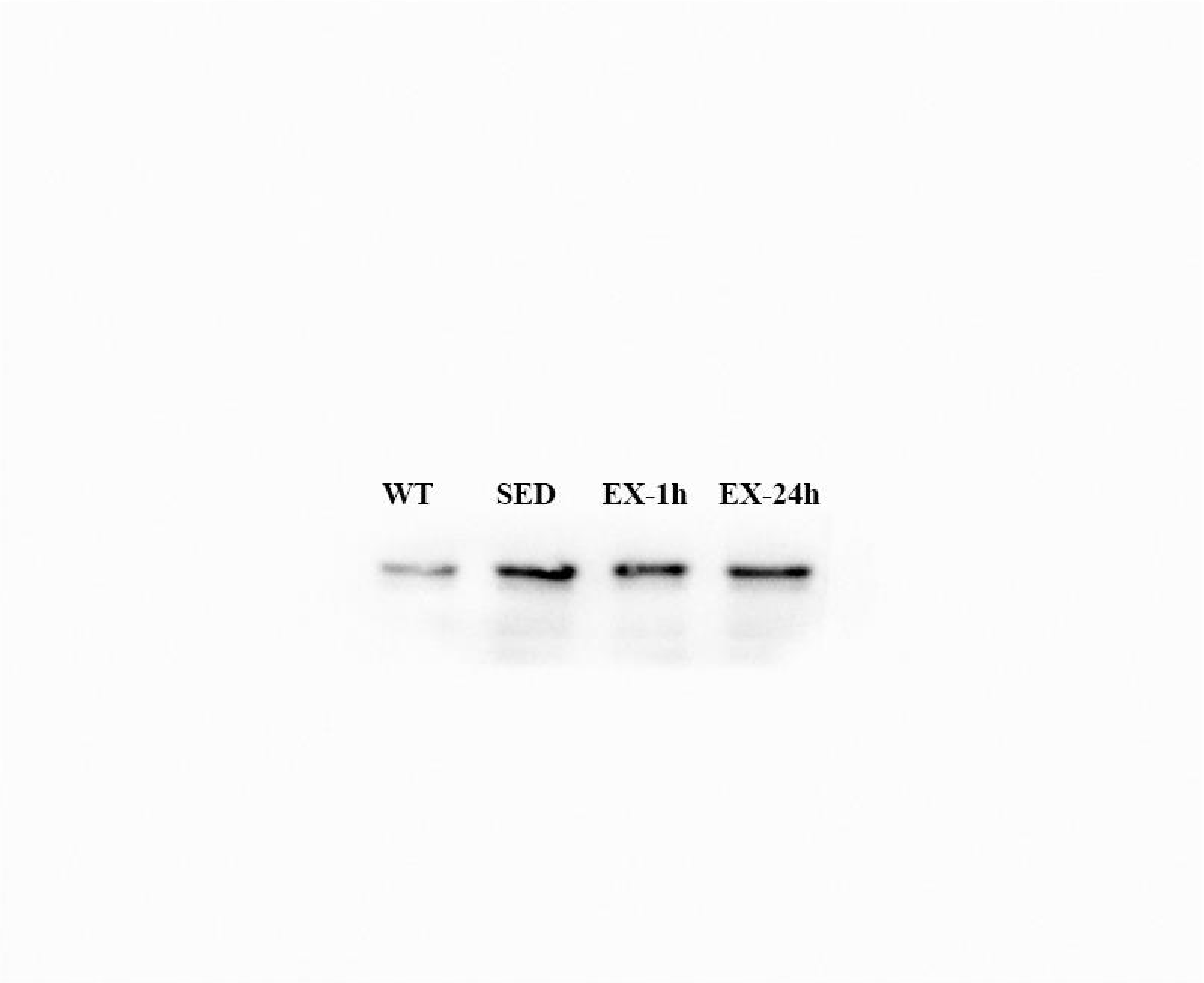

Sample 2, Western blot 3, IL-18, 23 kDa

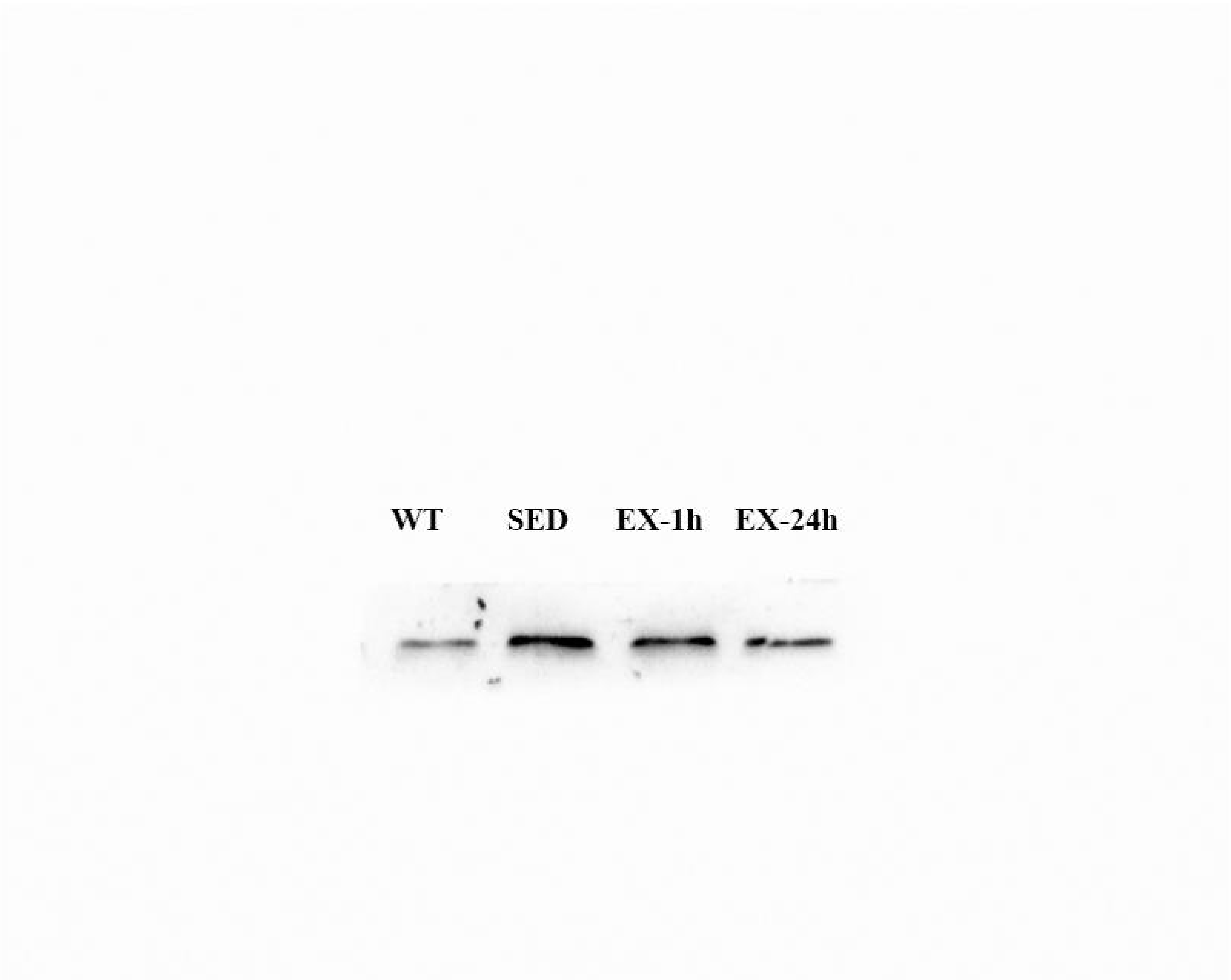

Sample 2, Western blot 3, GADPH, 36 kDa

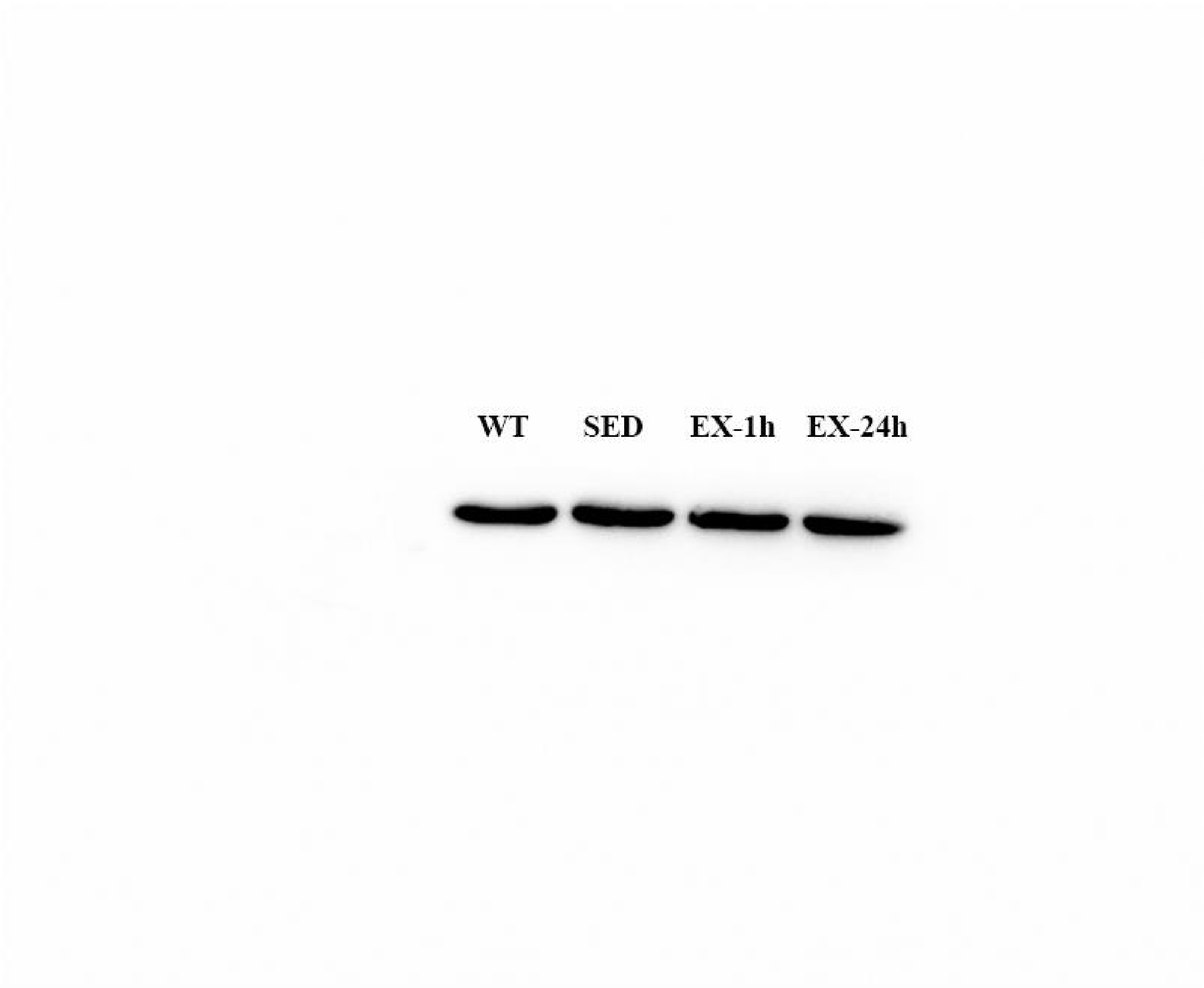

Supplement: S1 Raw images — (PDF) [file pone.0273527.s001.pdf]
